# Entinostat-Bortezomib Hybrids against Multiple Myeloma

**DOI:** 10.3390/molecules28031456

**Published:** 2023-02-02

**Authors:** Angelica Ferro, Dafni Graikioti, Emre Gezer, Constantinos M. Athanassopoulos, Muriel Cuendet

**Affiliations:** 1 School of Pharmaceutical Sciences, University of Geneva, 1211 Geneva, Switzerland; 2 Institute of Pharmaceutical Sciences of Western Switzerland, University of Geneva, 1211 Geneva, Switzerland; 3 Synthetic Organic Chemistry Laboratory, Department of Chemistry, University of Patras, 26504 Patras, Greece

**Keywords:** drug resistance, HDAC inhibitors, hybrid inhibitors, multiple myeloma, proteasome inhibitors

## Abstract

Although proteasome inhibitors have emerged as the therapeutic backbone of multiple myeloma treatment, patients often relapse and become drug refractory. The combination between proteasome and histone deacetylase inhibitors has shown to be more efficient compared to monotherapy by enhancing the anti-myeloma activity and improving the patient’s lifetime expectancy. Hybrid molecules, combining two drugs/pharmacophores in a single molecular entity, offer improved effectiveness by modulating more than one target and circumventing differences in the pharmacokinetic and pharmacodynamic profiles, which are the main disadvantages of combination therapy. Therefore, eleven histone deacetylase-proteasome inhibitor hybrids were synthesized, combining pharmacophores of entinostat and bortezomib. Compound **3** displayed the strongest antiproliferative activity with an IC_50_ value of 9.5 nM in the multiple myeloma cells RPMI 8226, 157.7 nM in the same cell line resistant to bortezomib, and 13.1 nM in a 3D spheroid model containing multiple myeloma and mesenchymal stem cells. Moreover, the compound inhibited 33% of histone deacetylase activity when RPMI 8226 cells were treated for 8 h at 10 µM. It also inhibited the proteasome activity with an IC_50_ value of 23.6 nM.

## 1. Introduction

Multiple myeloma (MM) is a hematological malignancy that occurs with an uncontrolled growth of malignant plasma cells, predominantly residing in the bone marrow [1,2]. It is the second most important blood disease following non-Hodgkin lymphoma, representing 10% among the hematological syndromes and accounting for 1% of all types of cancer [2]. It is generally preceded by asymptomatic phases termed monoclonal gammopathy of undetermined significance (MGUS) and smoldering multiple myeloma (SMM) [1,2]. SMM evolves to MM, which then presents well defined clinical manifestations such as hypercalcemia, kidney injury, anemia, bone lesions and an increased risk of infections [1,2]. Even though significant advances were made in the treatment options, MM patients are characterized by a history of relapses and drug resistance [3]. One of the main classes of anti-MM agents among the therapies of choice are proteasome inhibitors (PIs) [4] with the first-in-class bortezomib (BTZ) [5]. BTZ is a potent, selective and slowly reversible inhibitor of the β1 and β5 subunits of the 26S proteasome, the enzyme complex known as the intracellular machinery for protein degradation [5]. As a consequence, the accumulation of misfolded and unfolded proteins triggers various types of cellular stress responses, followed by the induction of apoptosis, inhibition of cell proliferation and cytotoxicity [5,6]. This also leads to the formation of aggregates, which activate the aggresome-autophagy pathway leading to protein clearing as a mechanism of resistance to treatment [5]. Histone deacetylase 3 (HDAC3) is another important target for the inhibition of MM proliferation. HDAC3 knockdown significantly induced apoptosis, and inhibitors showed synergistic effects with BTZ [7,8]. Therefore, combinations between PIs [9] and HDAC inhibitors (HDACi) [10,11], became a promising therapeutic strategy to address drug resistance in MM. Among the HDACi, entinostat (Ent), a class I selective HDACi, displayed synergistic antiproliferative activity when combined with BTZ in MM cells, including those resistant to BTZ [8,12]. Furthermore, the combination of the two inhibitors led to cell cycle arrest in G2/M phase and enhanced apoptosis [8,12].

The strategy of “hybrid molecules” has recently drawn increased attention in drug design and development, especially for the treatment of multifactorial diseases such as cancer [13]. This approach combines in one molecular entity the key structural features of various drugs, which are essential for their biological activity. This may result in a potent and selective hybrid molecule, able to activate two or more mechanisms of action toward cancer cell growth inhibition [14]. Compared to combination therapies, which involve the co-administration of multiple drugs, a hybrid molecule may provide certain advantages, such as improved pharmacokinetic properties, reduced side effects and a decrease in the development of drug resistance. Additionally, they may provide reduced costs and improved patient compliance, which is also of high importance [15].

**Figure 1:**
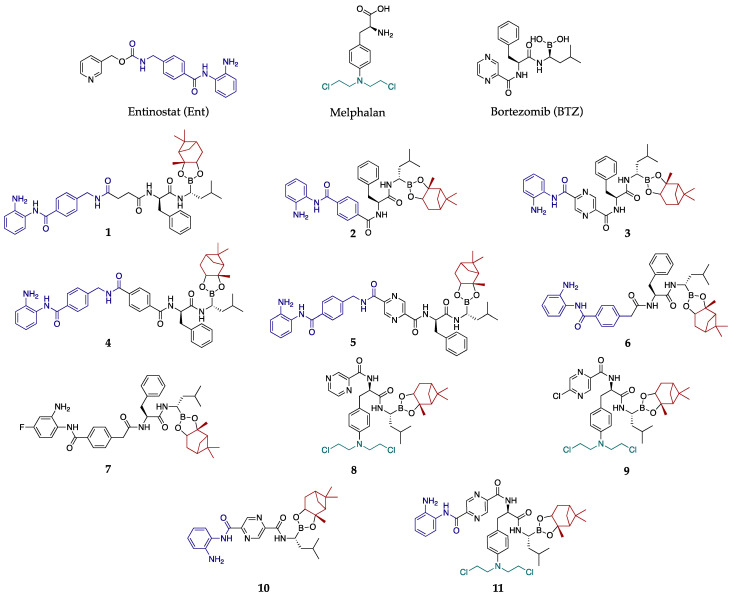
Structures of hybrid HDACi-BTZ compounds **1**–**11**.

As a result of our current interest in the development of novel therapeutic strategies to overcome resistance in MM, a library of 11 HDACi-BTZ hybrids, suitable for structure–activity relationship studies were synthesized (Figure 1). Based on the promising results of Zhou et al. [11] regarding similar dual inhibitors, the key pharmacophore structure of BTZ was kept. Thus, the dipeptide *L*-phenylalanine-*L*-boroleucine, including the pinanediol chiral auxiliary (as prodrug) was combined with various spacers and a zinc-binding group HDACi pharmacophore, such as the one of Ent. Moreover, an interesting set of compounds (**8**, **9**, **11**) was prepared, replacing the *L*-phenylalanine residue of the BTZ counterpart with melphalan, which combines the phenylalanine structure with a mustard type cytotoxic warhead.

## 2. Results and Discussion

### 2.1. Synthesis of Compounds ***1***–***11***

**Scheme 1:**
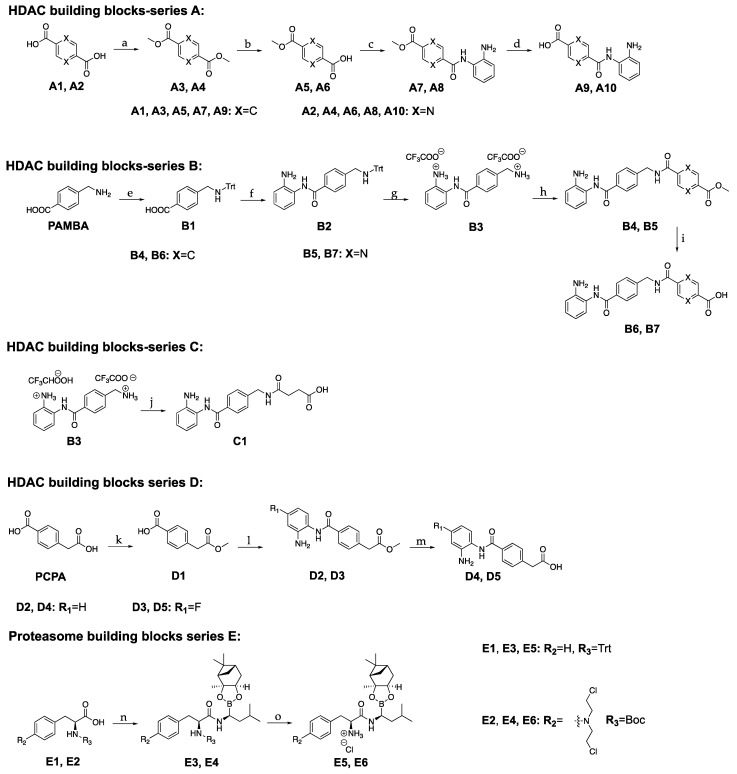
Synthetic pathways for the building blocks (**A**–**E**); Reagents and conditions: (**a**) SOCl_2_, CH_3_OH, reflux, 24 h, 36–88%; (**b**) NaOH, CH_3_OH, rt, 24 h, 97–98%; (**c**) 1,2-phenylenediamine, HBTU/Et_3_N or HATU/Et_3_N, DCM, 0 °C to rt, 24 h, 33–46%; (**d**) NaOH in CH_3_OH, DCM, rt, 3 h, 48%; (**e**) (CH_3_)_3_SiCl, Et_3_N, TrtCl, DCM/ACN, 0 °C to rt, 3 h, 68%; (**f**) 1,2-phenylenediamine, HATU/Et_3_N, DCM, 0 °C to rt, 24 h, 66%; (**g**) triethylsilane, TFA, DCM, 0 °C to rt, 1 h, 80%; (**h**) **A5** or **A6**, HBTU/Et_3_N, DMF or DCM, 0 °C to rt, 10–24 h, 44–89%; (**i**) LiOH in H_2_O, THF, rt, 3 h, 53% or NaOH in CH_3_OH, DCM, rt, 3 h, 86%; (**j**) succinic anhydride, DIPEA, THF/DMF, 0 °C to rt, 3 h, 82%; (**k**) (CH_3_)_3_SiCl, CH_3_OH, 0 °C to rt, 24 h, 60%; (**l**) 1,2-phenylenediamine or 4-fluoro-1,2-phenylenediamine, HBTU/Et_3_N, DMF, 0 °C to rt, 24 h, 30–36%; (**m**) NaOH in CH_3_OH, DCM, rt, 4 h, 66–75%; (**n**) (*R*)-boroleucine (1*S*,2*S*,3*R*,5*S*)-(+)-2,3-pinanediol ester trifluoroacetate, HATU/DIPEA, DMF, 0 °C to rt, 24 h, 68–70%; (**o**) trifluoroethanol, HCl in dioxane, DCM, 0 °C to rt, 2 h, 96–98%.

**Scheme 2:**
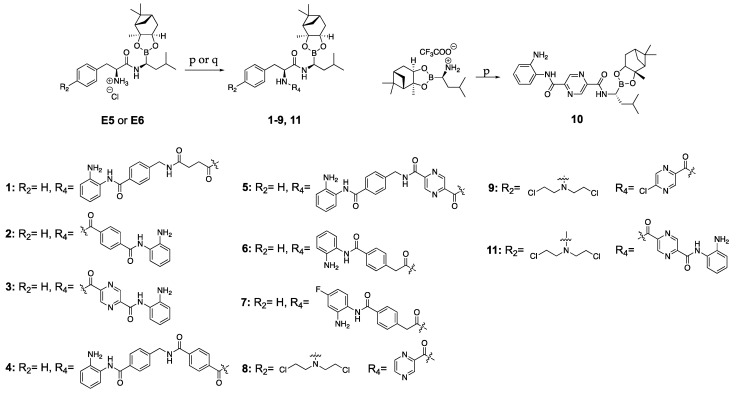
Synthetic pathways for compounds **1**–**11**; Reagents and conditions: (**p**) **A9** or **A10** or **B6** or **B7** or **D4** or **D5** or pyrazine-2-carboxylic acid or 5-chloropyrazine-2-carboxylic acid, TBTU/NMM, in DCM/DMF (1:2), (*R*)-boroleucine (1*S*,2*S*,3*R*,5*S*)-(+)-2,3-pinanediol ester trifluoroacetate or **E5** or **E6**, 0 °C to rt, 24 h, 38-63%; (**q**) **C1**, PyBOP/DIPEA, in DCM/DMF (1:2), **E5**, 0 °C to rt, 24 h, 60%.

The synthesis of the various building blocks and the final hybrids’ assembly is described in Scheme 1 and Scheme 2. The synthesis of the HDACi-spacer part involved a few reaction steps, starting from the commercially available terephthalic acid or pyrazine-2,5-dicarboxylic acid, or *p*-(aminomethyl)benzoic acid (PAMBA) or *p*-carboxyphenylacetic acid (PCPA). On the other hand, the BTZ part was built by coupling Boc-*L*-phenylalanine to *(R)*-Boro-leucine (1*S*,2*S*,3*R*,5*S*)-(+)-2,3-pinanediol ester trifluoroacetate.

The synthesis of the hybrid compounds (**1**–**11**) is described in Scheme 2 and was achieved by fragment coupling of the BTZ building block part (**E5** or **E6**) and the HDACi-spacer part (**A9** or **A10** or **B6** or **B7** or **D4** or **D5**), using typical amide coupling conditions (TBTU/NMM or PyBOP/DIPEA).

### 2.2. Antiproliferative Activity of Compounds ***1***–***11*** in Various MM Models

The antiproliferative potential of the compounds was assessed by measuring their metabolic activity in various MM models, and it was compared to BTZ and Ent. All of the compounds inhibited RPMI 8226 cell proliferation in a dose-dependent manner, except for compound **10**, which did not show any activity below 2000 nM (Table 1). This loss of activity can be attributed to the absence of phenylalanine residue in the BTZ part of the molecule.

Compound **3**, which incorporates the full structure of BTZ in its skeleton, was the most active with an IC_50_ value of 9.5 ± 1.4 nM which is only slightly inferior to BTZ (IC_50_ = 2.1 ± 0.4 nM). On the other hand, compound **2**, where the pyrazine-2,5-dicarboxylic acid moiety of the BTZ part was replaced by a terephthalic acid, was ca. 4 times less active with an IC_50_ value of 37.1 ± 2.9 nM. A similar decrease in activity was observed for the hybrid **5** (IC_50_ = 25.2 ± 2.2 nM)**,** which, compared to compound **3**, has one *p-*(aminomethyl)benzoyl group inserted between the pyrazine-2,5-dicarboxylic acid moiety and the 1,2-dianiline ring. The same structural change was applied to compound **2** to provide compound **4**. This led to a loss of activity (IC_50_ = 802.4 ± 72.7 nM). A similar activity was observed for compound **1** (IC_50_ = 677.7 ± 42.5 nM), in which the terephthalyl moiety of hybrid **4** was replaced by a succinyl one. Moreover, compound **11**, which, compared to compound **3**, has the phenylalanine moiety replaced by melphalan, showed ca 2.4 times less activity than **3**. In addition, compounds **8** and **9,** bearing melphalan’s warhead and having a structural similarity with BTZ, exhibited lower activity than BTZ itself. The above results indicate that the insertion of a cytotoxic warhead to BTZ or hybrid **3** structures did not offer any improvement of activity compared to BTZ.

The frequent occurrence of drug resistance in MM patients is one of the reasons for the need to identify new active molecules. Therefore, compounds that showed an IC_50_ value below 50 nM in RPMI 8226 cells were tested in RPMI 8226 cells resistant to BTZ (RPMI 8226/BTZ100) to evaluate if the hybrids could overcome PI resistance (Table 1). The results demonstrated that compounds **2**, **3**, and **5** were more potent than BTZ against RPMI 8226/BTZ100 cells. Moreover, the increased resistance was different depending on the compounds and corresponded to 3.0, 16.6, and 8.0-fold for compounds **2**, **3**, and **5**, respectively, compared to 116.8-fold for BTZ. Furthermore, some compounds that had similar IC_50_ values in RPMI 8226 cells showed considerable differences in their activity in the resistant cells (compounds **2** and **7**).

The interactions between MM cells and the bone marrow microenvironment play an important role in MM progression. The culture of a single cell line as a monolayer attached to a plastic surface does not properly represent the tumor complexity and interactions with the extracellular compartment. Therefore, a 3D co-culture spheroid model containing RPMI 8226 cells and mesenchymal stem cells (MSC) in a 5:1 ratio was used to measure the antiproliferative activity of compounds that showed an IC_50_ value inferior to 50 nM in RPMI 8226 cells (Table 1). Compound **3** showed the strongest activity with an IC_50_ value of 13.1 ± 6.9 nM. For the most active compounds, the activity remained the same or was slightly lower in the spheroids than in RPMI 8226 cells. This showed that those compounds kept their activity in a more complex model.

### 2.3. 26S Proteasome Inhibitory Activity of Compounds ***1***–***11***

To evaluate whether the proteasome inhibitory activity of BTZ was maintained in the complex molecular structure of the hybrids, each compound was tested for its capacity to inhibit the 26S proteasome in RPMI 8226 cell lysate and the IC_50_ was measured when possible (Table 1). The inhibitory activity of compounds **1**, **2**, **7**, and **8** was similar to BTZ, while compounds **3** and **5** were slightly less active. In general, all of the above-mentioned compounds maintained a very good PI activity in the low nM range, although their antiproliferative activity against RPMI 8226 cells may differ and follow a different order.

### 2.4. HDAC Inhibitory Activity in RPMI 8226 Cells 

To evaluate whether the HDAC inhibitory activity of Ent was maintained in the complex molecular structure of the hybrids, the activity of compounds inhibiting RPMI 8226 cell proliferation with an IC_50_ value lower than 50 nM was tested in the same cell line using a UHPLC-MS method. Compounds **2**, **3**, **7**, and **11** inhibited 50, 33, 37, and 25%, respectively, of HDAC activity at 10 µM. Ent, on the other hand, inhibited 51% of HDAC activity at 10 µM and showed an IC_50_ value of 9.3 ± 0.7 µM. Data indicated that compound **2** and Ent had a similar activity at 10 µM. However, it was not possible to measure the IC_50_ value of the hybrids due to poor water solubility.

### 2.5. Drug Combination

The antiproliferative effect of Ent and BTZ in combination at various concentrations was evaluated to determine if there was any synergy between both compounds. Combinations were evaluated using Combenefit data analysis. Ent (20–1600 nM) and BTZ (0.3–20 nM) did not show any synergistic effects when tested around their IC_50_ values nor when tested in a 1:1 ratio (0.3–20 nM). However, when the drug combination in a 1:1 ratio was analyzed using the CompuSyn software, Ent and BTZ displayed synergistic effects when combined at 5 nM (CI = 0.59) with 91% cell proliferation inhibition. Despite having a CI < 1 indicating synergy, the IC_50_ values between BTZ and the drug combination did not differ significantly (3.3 vs. 3.2 nM), suggesting that the addition of Ent at low doses did not improve the biological activity of BTZ.

## 3. Materials and Methods

### 3.1. General Methods

All solvents were dried and purified according to standard procedures prior to use. When required, reactions were performed under inert atmosphere (Ar) in pre-flamed glassware. Anhydrous Na_2_SO_4_ was used for drying solutions, and the solvents were then routinely removed at ca. 40 °C under reduced pressure using a rotary vacuum evaporator. All reagents employed in the present work were commercially available and used without further purification. Flash column chromatography (FCC) was performed on silica gel (70–230 and 230–400 mesh, Merck, Darmstadt, Germany) and analytical thin layer chromatography (TLC) on silica gel 60-F254 precoated aluminum foils (0.2 mm film, Merck, Germany). Spots on the TLC plates were visualized with UV light at 254 nm and using ninhydrin solution. ^1^H NMR spectra were recorded in CDCl_3_ at 600.13 MHz and ^13^C spectra at 150.9 MHz on a Bruker AVANCEIII HD spectrometer. Chemical shifts (*δ*) are indicated in parts per million downfield from TMS and coupling constants (*J*) are reported in Hz. Copies of selected ^1^H and ^13^C NMR spectra can be found at Supplementary Materials. ESI mass spectra were recorded at 30 V, on a Micromass-Platform LC spectrometer using MeOH as solvent. HR mass spectra were performed using a Bruker AUTOFLEX SPEED MALDI-TOF/TOF or a Bruker Maxis Impact QTOF Spectrometer. FT-IR spectra (4000–400 cm^−1^) were recorded using a Thermo Scientific Nicolet iS20 spectrometer with samples prepared as KBr pellets.

### 3.2. Synthesis

Dimethyl pyrazine-2,5-dicarboxylate (**A4**): To a suspension of pyrazine-2,5-dicarboxylic acid (3.15 g, 18.75 mmol) in anhydrous MeOH (40 mL), cooled at 0 °C. SOCl_2_ (1 mL, 13.8 mmol) was added dropwise, and the mixture was heated at 80 °C for 24 h. Upon completion, the suspension was filtered while hot and the filtrate was concentrated to dryness. The desired diester was afforded as pale-yellow fluffy crystals (1.08 g, 36% yield) after FCC purification using toluene/ethyl acetate 7:3 as eluent. R*f* (PhMe/EtOAc 7:3) = 0.25; ^1^H NMR (CDCl_3_, 600.13 MHz) δ 9.39 (s, 2 H), 4.07 (s, 6 H); ^13^C NMR (CDCl_3_, 150.9 MHz) δ 163.5, 145.5, 145.2, 53.5.

5-(methoxycarbonyl)pyrazine-2-carboxylic acid (**A6**): Into a suspension of dimethyl pyrazine-2,5-dicarboxylate) (0.3 g, 1.53 mmol) in MeOH (38.2 mL), 1 M NaOH in water (1.53 mmol) was dripped and the solution was stirred at room temperature for 24 h [16]. The resulting mixture was concentrated to dryness under vacuum, and the residue was dissolved in water (10 mL). Then HCl_(conc.)_ (~5 mL) was dripped into the solution until a pale-yellow solid was precipitated. The suspension was filtrated under vacuum and air dried to give the acid as a pale-yellow solid (0.250 g, 98% yield), without further purification. R*f* (DCE/MeOH/CH_3_COOH) 8:2:0.5 = 0.2; MS (ESI, 30 eV): *m*/*z* 181.2 [M-H^+^].

Methyl 5-((2-aminophenyl)carbamoyl)pyrazine-2-carboxylate (**A8**): To a stirring suspension of 5-(methoxycarbonyl)pyrazine-2-carboxylic acid (435 mg, 2.39 mmol) in anhydrous DCM (3 mL), Et_3_N (1.15 mL, 8.24 mmol) and HBTU (1.15 g, 3.03 mmol) were added and the mixture was cooled at 0 °C. Then, 1,2-phenylenediamine (890 mg, 8.24 mmol) was added and the mixture was stirred at room temperature for 24 h. Then the solvent was evaporated under reduced pressure, and the desired amide was afforded as a yellow solid (300 mg, 46% yield) after recrystallization of the dark brown residue, from toluene. R*f* (PhMe/EtOAc) 1:1 = 0.12; MS (ESI, 30 eV): *m*/*z* 273.27 [M+H^+^], 295.51 [M+Na^+^]; ^1^H NMR (CDCl_3_, 600.13 MHz) δ 9.59 (d, *J* = 1.2 Hz, 2 H), 9.30 (d, *J* = 1.2 Hz, 1 H), 7.52 (dd, *J* = 1.8 Hz, *J*′ = 7.8 Hz, 1 H), 7.12 (td, *J* = 1.8 Hz, *J*′ = 7.8 Hz, 1 H), 6.88 (ddd, *J* =1.2 Hz, *J*′ = 7.8 Hz, *J*″ = 7.2 Hz, *J*‴ = 14.4 Hz, 2 H), 4.09 (s, 3 H); ^13^C NMR (CDCl_3_, 150.9 MHz) δ 163.8, 159.9, 146.3, 145.3, 144.1, 144.0, 140.0, 127.4, 124.4, 123.7, 120.0, 118.5, 53.5. 

5-((2-aminophenyl)carbamoyl)pyrazine-2-carboxylic acid (**A10**): Into a stirring yellow suspension of methyl 5-((2-aminophenyl)carbamoyl)pyrazine-2-carboxylate (220 mg, 0.81 mmol) in DCM (4 mL), 3 M NaOH in MeOH (0.8 mL, 2.42 mmol) was dripped and the dark red solution was stirred at room temperature for 3 h. Upon consumption of the starting material the mixture was concentrated under vacuum and the residue was acidified with 5% citric acid_(aq)_ up to pH 3.5-4. Then the mixture was concentrated to dryness and the desired acid was afforded as dark brown solid (100 mg, 48% yield) without further purification. R*f* (DCE/MeOH/CH_3_COOH) 8:2:1 = 0.28; MS (ESI, 30 eV): *m*/*z* 257.34 [M-H^+^].

Dimethyl terephthalate (**A3**): Suspension of terephthalic acid (4 g, 24.07 mmol) in MeOH (200 mL) was heated under reflux for 2 h [17]. Then the suspension was cooled at 0 °C and SOCl_2_ (50 mL, 485 mmol) was dripped carefully into the system. The reaction mixture was stirred at 80 °C for 24 h. The resulting solution was evaporated to dryness and the residue was subjected to FCC purification using toluene/ethyl acetate 8:2 as eluent, to give the desired diester as white fluffy crystals (4.1 g, 88% yield). R*f* (PhMe/EtOAc) 8:2 = 0.56; ^1^H NMR (CDCl_3_, 600.13 MHz) δ 8.10 (s, 4 H), 3.94 (s, 6 H); ^13^C NMR (CDCl_3_, 150.9 MHz) δ 166.3, 133.9, 129.5, 52.4. 

4-(methoxycarbonyl)benzoic acid (**A5**): Into a suspension of dimethyl terephthalate (2 g, 10.29 mmol) in MeOH (257.2 mL), 1 M NaOH in water (10.30 mmol) was dripped and the solution was stirred at room temperature for 24 h. The resulting mixture was concentrated to dryness under vacuum, and the residue was dissolved in water (~50 mL). Then HCl_(conc.)_ (~70 mL) was dripped into the solution until a white solid was precipitated. The suspension was filtered under vacuum and air dried to give the acid as a white solid (1.8 g, 97% yield), without further purification. R*f* (EtOAc) = 0.1.

Methyl 4-((2-aminophenyl)carbamoyl)benzoate (**A7**): To a stirring suspension of 4-(methoxycarbonyl)benzoic acid (400 mg, 2.22 mmol) in anhydrous DCM (3.2 mL), Et_3_N (560 μL, 4 mmol) and HATU (950 mg, 2.5 mmol) were added and the mixture was cooled at 0 °C. Then, 1,2-phenylenediamine (540 mg, 5 mmol) was added and the mixture was stirred at room temperature for 24 h. Upon completion, the mixture was concentrated to dryness and the residue was subjected to FCC purification using toluene/ethyl acetate 8:2 as eluent. The desired amide was afforded as pale-white solid (192 mg, 33% yield). R*f* (PhMe/EtOAc) 8:2 = 0.16; MS (ESI, 30 eV): *m*/*z* 271.38 [M+H^+^], 293.30 [M+Na^+^]; ^1^H NMR (CDCl_3_, 600.13 MHz) δ 8.15 (d, *J* = 7.8 Hz, 2 H), 7.97 (d, *J* = 7.8 Hz, 2 H), 7.92 (s, 1 H), 7.37 (d, *J* = 7.8 Hz, 1 H), 7.11 (t, *J* = 7.8 Hz, 1 H), 6.87 (d, *J* = 7.8 Hz, 2 H), 3 96 (s, 3 H); ^13^C NMR (CDCl_3_, 150.9 MHz) δ 166.2, 140.4, 130.0, 127.3, 120.0, 118.7, 52.5.

4-((2-aminophenyl)carbamoyl)benzoic acid (**A9**): Into a stirring suspension of methyl 4-((2-aminophenyl)carbamoyl)benzoate (240 mg, 0.88 mmol) in DCM (4.4 mL), 3 M NaOH in MeOH (0.88 mL, 2.64 mmol) was dripped and the dark yellow solution was stirred at room temperature for 3 h [18]. Upon consumption of the starting material, the mixture was concentrated under vacuum and the residue was acidified with 5% citric acid_(aq)_ up to pH 4. Then the mixture was placed in a separatory funnel and extracted four times with DCM. The combined organic layers were washed with brine, dried over anhydrous Na_2_SO_4_ and concentrated to dryness under vacuum. The desired acid was afforded as pale-yellow solid (107 mg, 48% yield) without further purification. R*f* (DCE/MeOH/CH_3_COOH) 8:2:1 = 0.66; MS (ESI, 30 eV): *m*/*z* 255.20 [M-H^+^]; ^1^H NMR (CDCl_3_, 600.13 MHz) δ 8.15–8.19 (m, 4 H), 7.48–7.52 (m, 4 H); ^13^C NMR (CDCl_3_, 150.9 MHz) δ 167.3, 134.0, 129.5, 128.7, 127.9, 127.6, 126.4, 123.6. 

4-((tritylamino)methyl)benzoic acid (**B1**): To a stirring suspension of 4-(aminomethyl) benzoic acid (4.53 g, 30 mmol) in a mixture of anhydrous DCM (52.5 mL) and ACN (7.5 mL), trimethylchlorosilane (4.19 mL, 33 mmol) was added, and the resulting mixture was heated under reflux for 30 min. After cooling at room temperature, the solution was cooled at 0 °C and triethylamine (17.5 mL, 120 mmol) was added dropwise into the system, followed by a portion-wise addition of TrtCl (8.78 g, 31.5 mmol) within 30 min. The resulting white emulsion was stirred vigorously for 1 h at 0 °C, and additionally for 3 h at room temperature. Then, the mixture was cooled at 0 °C, MeOH (3 mL) was added and after 30 min the mixture was concentrated to dryness. The resulting residue was diluted in 4 M NaOH_(aq)_ (30 mL), placed in a separatory funnel, and twice extracted with diethyl ether. The aqueous layer was acidified with ice-cooled 5% citric acid_(aq)_ up to pH 4–5 and twice extracted with ethyl acetate. The combined organic layers were, thereupon, washed with water and brine, dried over anhydrous Na_2_SO_4_, filtered and concentrated to dryness to afford the product as white foam (8 g, 68% yield) without further purification. R*f* (PhMe/EtOAc) 8:2= 0.27; MS (ESI, 30 eV): *m*/*z* 392.32 [M-H^+^].

*N*-(2-aminophenyl)-4-((tritylamino)methyl)benzamide (**B2**): To a stirring suspension of 4-((tritylamino)methyl) benzoic acid (1 g, 2.54 mmol) in anhydrous DCM (3.6 mL), Et_3_N (1.3 mL, 10 mmol) and HATU (988 mg, 2.6 mmol) were added and the mixture was cooled at 0 °C. Then, 1,2-phenylenediamine (325 mg, 3 mmol) was added and the mixture was stirred at room temperature for 24 h. Upon completion, the mixture was placed in a separatory funnel and washed with ice-cooled 5% citric acid_(aq)_, water and brine. The organic layer was dried over anhydrous Na_2_SO_4,_ filtered and concentrated to dryness. The residue was subjected to FCC purification using toluene/ethyl acetate 9:1 as eluent and the desired amide was afforded as white crystalline solid (812 mg, 66% yield). R*f* (PhMe/EtOAc) 9:1 = 0.23; MS (ESI, 30 eV): *m*/*z* 243.91 [Trt^+^], 506.73 [M+Na^+^], 989.60 [2M+Na^+^]; ^1^H NMR (CDCl_3_, 600.13 MHz) δ 7.87 (d, *J* = 7.8 Hz, 2 H), 7.56 (d, *J* = 7.8 Hz, 5 H), 7.53 (d, *J* = 8.4 Hz, 2 H), 7.31 (t, *J* = 7.8 Hz, 7 H), 7.23 (t, *J* = 7.2 Hz, 3 H), 7.10 (t, *J* = 7.2 Hz, 1 H), 6.85–6.89 (m, 2 H), 3.44 (s, 2 H); ^13^C NMR (CDCl_3_, 150.9 MHz) δ 129.0, 128.6, 128.2, 128.0, 127.4, 126.6, 125.3, 47.7.

Bis (2-(4-(ammoniomethyl)benzamido)benzenaminium)) 2,2,2-trifluoroacetate salt (**B3**): Triethylsilane (380 μL, 2.37 mmol) and trifluoroacetic acid (386 μL, 5.04 mmol) were dripped into an ice-cooled solution of *N*-(2-aminophenyl)-4-((tritylamino)methyl) benzamide (812 mg, 1.68 mmol) in anhydrous DCM (3.5 mL) and the resulting mixture was initially stirred at 0 °C and then at room temperature for 1 h. Upon consumption of the starting material, concentration of the solution up to 0.2 mL took place, followed by the successive dropwise addition of diethyl ether and hexane, respectively. The white precipitate was twice washed with hexane and the desired salt was afforded as white amorphous solid (623 mg, 80% yield). MS (ESI, 30 eV): *m*/*z* 242.41 [M+H^+^], 264.39 [M+Na^+^].

Methyl 4-((4-((2-aminophenyl)carbamoyl)benzyl)carbamoyl)benzoate (**B4**): Bis (2-(4-(ammoniomethyl)benzamido)benzenaminium) 2,2,2-trifluoroacetate salt (105 mg, 0.22 mmol) and 4-(methoxycarbonyl)benzoic acid (41 mg, 0.23 mmol) were dissolved in anhydrous DMF (0.23 mL) and the solution was cooled at 0 °C. Then the addition of HBTU (102 mg, 0.27 mmol) took place, followed by dropwise addition of Et_3_N (94 μL, 0.68 mmol). The reaction mixture was initially stirred at 0 °C and then at room temperature for 10 h. Then, the solution was diluted in DCM and twice successively washed with water, 5% NaHCO_3(aq)_ solution, and brine. The organic layer was dried over anhydrous Na_2_SO_4_, filtered, and concentrated to dryness to afford an oily residue. The desired amide was afforded as a pale-white solid (40 mg, 44% yield) after FCC purification of the residue using toluene/ethyl acetate 9:1 as eluent. R*f* (PhMe/EtOAc) 9:1 = 0.2; MS (ESI, 30 eV): *m*/*z* 426.26 [M+Na^+^], 442.33 [M+K^+^]; ^1^H NMR (CDCl_3_, 600.13 MHz) δ 8.07–8.11 (m, 2 H), 7.85–7.89 (m, 2 H), 7.73–7.75 (m, 1 H), 7.44–7.46 (m, 1 H), 7.32–7.36 (m, 4 H), 7.01–7.04 (m, 2 H), 6.80–6.82 (m, 1 H), 5.07 (d, *J* = 1.8 Hz, 1 H), 4.66 (d, *J* = 5.4 Hz, 1 H), 3.94 (s, 3 H); ^13^C NMR (CDCl_3_, 150.9 MHz) δ 129.9, 128.5, 127.1, 126.7, 38.6.

4-((4-((2-aminophenyl)carbamoyl)benzyl)carbamoyl)benzoic acid (**B6**): 2 M LiOH in water (0.2 mL, 0.4 mmol) was dripped into a stirring suspension of methyl 4-((4-((2-aminophenyl)carbamoyl)benzyl)carbamoyl)benzoate (40 mg, 0.1 mmol) in THF (0.7 mL) and the dark yellow solution was stirred at room temperature for 3 h. Upon consumption of the starting material, the mixture was concentrated under vacuum and the residue was acidified with 5% citric acid_(aq)_ up to pH 4. Then the mixture was placed in a separatory funnel and extracted four times with DCM. The combined organic layers were washed with brine, dried over anhydrous Na_2_SO_4_ and were concentrated to dryness under vacuum. The desired acid was afforded as pale-yellow solid (20 mg, 53% yield) without further purification. 

Methyl 5-((4-((2-aminophenyl)carbamoyl)benzyl)carbamoyl)pyrazine-2-carboxylate (**B5**): Bis (2-(4-(ammoniomethyl)benzamido)benzenaminium) 2,2,2-trifluoroacetate salt (270 mg, 0.57 mmol) and 5-(methoxycarbonyl)pyrazine-2-carboxylic acid (100 mg, 0.55 mmol) were dissolved in anhydrous DCM (0.72 mL) and the solution was cooled at 0 °C. Then, the addition of HBTU (230 mg, 0.61 mmol) took place, followed by dropwise addition of Et_3_N (307 μL, 2.2 mmol). The reaction mixture was initially stirred at 0 °C and then at room temperature for 24 h. The desired amide was afforded as a yellow solid (200 mg, 89% yield) after recrystallization of the dark brown mixture, from toluene. R*f* (PhMe/EtOAc) 9:1 = 0.38; MS (ESI, 30 eV): *m*/*z* 428.28 [M+Na^+^]; ^1^H NMR (CDCl_3_, 600.13 MHz) δ 9.53–9.54 (m, 1 H), 9.23–9.24 (m, 1 H), 8.25–8.26 (m, 1 H), 7.96 (d, *J* = 7.8 Hz, 1 H), 7.89 (d, *J* = 7.8 Hz, 1 H), 7.77 (d, *J* = 7.8 Hz, 1 H), 7.56 (d, *J* = 7.8 Hz, 1 H), 7.47 (d, *J* = 7.8 Hz, 1 H), 7.44 (d, *J* = 7.8 Hz, 1 H), 7.33–7.34 (m, 1 H), 7.08 (t, *J* = 7.2 Hz, 1 H), 6.87 (t, *J* = 7.8 Hz, 1 H), 4.75–4.76 (m, 1 H), 4.07 (s, 3 H); ^13^C NMR (CDCl_3_, 150.9 MHz) δ 163.8, 162.2, 146.1, 145.8, 145.6, 145.3, 144.4, 144.1, 144.0, 143.9, 141.8, 133.5, 128.0, 127.9, 127.3, 126.4, 124.7, 53.4, 43.2.

5-((4-((2-aminophenyl)carbamoyl)benzyl)carbamoyl)pyrazine-2-carboxylic acid (**B7**): 3 M NaOH in MeOH (0.74 mL, 2.22 mmol) was dripped into a stirring suspension of methyl 5-((4-((2-aminophenyl)carbamoyl)benzyl)carbamoyl)pyrazine-2-carboxylate (300 mg, 0.74 mmol) in DCM (3.7 mL) and the dark red solution was stirred at room temperature for 3 h. Upon consumption of the starting material, the mixture was concentrated under vacuum and the residue was acidified with 5% citric acid_(aq)_ solution up to pH 3.5. Then the mixture was concentrated to dryness and the desired acid was afforded as pale-yellow solid (250 mg, 86% yield) without further purification. R*f* (DCE/MeOH/CH_3_COOH) 8:2:0.5 = 0.3.

4-((4-((2-aminophenyl)carbamoyl)benzyl)amino)-4-oxobutanoic acid (**C1**): DIPEA (50 μL, 0.26 mmol) was dripped into a stirring solution of bis (2-(4-(ammoniomethyl)benzamido)benzenaminium) 2,2,2-trifluoroacetate salt (45 mg, 0.10 mmol) in a mixture of THF (400 μL) and DMF (100 μL) and the resulting solution was cooled at 0 °C. Then, succinic anhydride (120 mg, 1.2 mmol) was added, and the reaction mixture was stirred for 3 h at room temperature. Subsequently, the mixture was subjected to FCC purification using dichloromethane/methanol/acetic acid 85:10:5 as eluent, and the desired acid was afforded as pale-yellow oil (28 mg, 82% yield). R*f* (DCE/MeOH/CH_3_COOH) 85:15:0.1 = 0.08; MS (ESI, 30 eV): *m*/*z* 364.65 [M+Na^+^]; ^1^H NMR (CDCl_3_, 600.13 MHz) δ 9.50 (s, 1 H), 8.07 (d, *J* = 8.4 Hz, 1 H), 7.87 (d, *J* = 7.8 Hz, 2 H), 7.55 (brs, 1 H), 7.34 (d, *J* = 7.8 Hz, 2 H), 7.16 (dd, *J*′ = 7.8, *J*″ = 1.8 Hz, 1 H), 7.11–7.12 (m, 1 H), 4.46 (d, *J* = 6 Hz, 2 H), 2.70 (dd, *J*′ = 7.8, *J*″ = 4.8 Hz, 2 H), 2.61–2.65 (m, 4 H), 2.52–2.54 (m, 2 H); ^13^C NMR (CDCl_3_, 150.9 MHz) δ 175.0, 133.0, 127.7, 127.3, 125.0, 52.0, 40.9, 31.8.

4-(2-methoxy-2-oxoethyl) benzoic acid (**D1**): 4-(carboxymethyl) benzoic acid (200 mg, 1.11 mmol) was added in MeOH (2.22 mL) and the suspension was cooled at 0 °C. Then, trimethylchlorosilane (10 μL, 0.05 mmol) was dripped into the mixture and the resulting solution was initially stirred at 0 °C and then at room temperature for 24 h. Upon completion of the reaction, the mixture was concentrated under vacuum to dryness, and diethyl ether was added to the residue. The suspension was thrice successively washed with 5% NaHCO_3(aq)_ and water, and the combined aqueous layers were acidified with 1 M HCl_(aq)_ up to the total precipitation of the white solid. The aqueous mixture was extracted five times with diethyl ether and the organic layers were washed with water and brine, dried over anhydrous Na_2_SO_4_ and concentrated to dryness under vacuum to afford the desired ester (130 mg, 60% yield) as colorless oil. R*f* (DCM/MeOH) 9:1 = 0.45; MS (ESI, 30 eV): *m*/*z* 193.38 [M-H^+^]; ^1^H NMR (CDCl_3_, 600.13 MHz) δ 8.08 (d, *J* = 8.4 Hz, 2 H), 7.40 (d, *J* = 8.4 Hz, 2 H), 3.72 (s, 3 H), 3.71 (s, 2 H); ^13^C NMR (CDCl_3_, 150.9 MHz) δ 171.6, 171.2, 140.1, 130.5, 129.5, 128.2, 52.3, 41.2.

Methyl 2-(4-((2-aminophenyl)carbamoyl)phenyl)acetate (**D2**): To a stirring solution of 4-(2-methoxy-2-oxoethyl) benzoic acid (60 mg, 0.31 mmol) in anhydrous DMF (0.44 mL), Et_3_N (167 μL, 1.2 mmol) and HBTU (152 mg, 0.4 mmol) were added and the mixture was cooled at 0 °C. Then, 1,2-phenylenediamine (44 mg, 0.4 mmol) was added and the mixture was stirred at room temperature for 24 h. Upon completion, the mixture was diluted in DCM and twice successively washed with water, ice-cooled 5% NaHCO_3(aq)_, water and brine. The organic layer was dried over anhydrous Na_2_SO_4_, filtered and concentrated to dryness under vacuum to afford an oily residue. The desired amide was afforded as a pale-yellow solid (32 mg, 36% yield) after FCC purification using toluene/ethyl acetate 6:4 as eluent. R*f* (PhMe/EtOAc) 6:4 = 0.26; MS (ESI, 30 eV): *m*/*z* 258.49 [M+H^+^], 307.22 [M+Na^+^]; ^1^H NMR (CDCl_3_, 600.13 MHz) δ 8.10 (s, 1 H), 7.83 (d, *J* = 7.8 Hz, 2 H), 7.35 (d, *J* = 7.8 Hz, 2 H), 7.28 (d, *J* = 8.4 Hz, 1 H), 7.06 (td, *J* = 7.8, *J*′ = 1.8 Hz, 1 H), 6.81–6.83 (m, 2 H), 3.71 (s, 3 H), 3.69 (s, 2 H); ^13^C NMR (CDCl_3_, 150.9 MHz) δ 171.4, 165.6, 140.6, 138.0, 133.0, 129.7, 127.6, 127.2, 125.3, 124.6, 124.6, 119.9, 119.8, 118.4, 52.2, 40.9.

2-(4-((2-aminophenyl)carbamoyl)phenyl)acetic acid (**D4**): 3 M NaOH in MeOH (0.35 mL, 1.05 mmol) was dripped into a stirring solution of methyl 2-(4-((2-aminophenyl)carbamoyl)phenyl)acetate (100 mg, 0.35 mmol) in DCM (1.75 mL) and the mixture was stirred at room temperature for 4 h. Upon consumption of the starting material, the mixture was concentrated under vacuum and the residue was acidified with 5% citric acid_(aq)_ up to pH 4. The precipitated solid was diluted in methanol and the resulting mixture was then filtered and concentrated to dryness to afford the product as a pale-yellow oil, without further purification. (62 mg, 66% yield). R*f* (DCE/MeOH/CH_3_COOH) 8:2:1 = 0.62.

Methyl 2-(4-((2-amino-4-fluorophenyl)carbamoyl)phenyl)acetate (**D3**): To a stirring solution of 4-(2-methoxy-2-oxoethyl) benzoic acid (42 mg, 0.22 mmol) in anhydrous DMF (0.31 mL), Et_3_N (92 μL, 0.66 mmol) and HBTU (114 mg, 0.3 mmol) were added and the mixture was cooled at 0 °C. Then, 1,2-diamino 4-fluorobenzene (38 mg, 0.3 mmol) was added and the mixture was stirred at room temperature for 24 h. Upon completion, the mixture was diluted in DCM and twice successively washed with water, ice-cooled 5% NaHCO_3(aq)_, water and brine. The organic layer was dried over anhydrous Na_2_SO_4_, filtered, and concentrated to dryness under vacuum to afford an oily residue. The desired amide was afforded as a pale-yellow solid (19.2 mg, 30% yield) after FCC purification using toluene/ethyl acetate 6:4 as eluent. R*f* (PhMe/EtOAc) 6:4 = 0.49; ^1^H NMR (CDCl_3_, 600.13 MHz) δ 7.88 (d, *J* = 7.8 Hz, 2 H), 7.40 (s, 2 H), 7.20 (s, 1 H), 6.57 (s, 2 H), 3.73 (s, 3 H), 3.71 (s, 2 H); ^13^C NMR (CDCl_3_, 150.9 MHz) δ 175.1, 148.0, 146.8, 137.4, 129.8, 129.7, 52.2, 40.9, 25.6.

2-(4-((2-amino-4-fluorophenyl)carbamoyl)phenyl) acetic acid (**D5**): 3 M NaOH in MeOH (0.29 mL, 0.85 mmol) was dripped into a stirring solution of methyl 2-(4-((2-amino-4-fluorophenyl)carbamoyl)phenyl)acetate (86 mg, 0.29 mmol) in DCM (1.49 mL) and the mixture was stirred at room temperature for 4 h. Upon consumption of the starting material, the mixture was concentrated under vacuum and the residue was acidified with 5% citric acid_(aq)_ up to pH 3-4. The precipitated solid was diluted in methanol and the resulting mixture was then filtered and concentrated to dryness to afford the product as a pale-yellow solid, without further purification. (62 mg, 75% yield). R*f* (DCE/MeOH/CH_3_COOH) 8:2:1 = 0.53.

(*S*)-3-(4-(bis(2-chloroethyl)amino)phenyl)-2-((*tert*-butoxycarbonyl)amino) propanoic acid (**E2**): To a suspension of melphalan (30 mg, 0.1 mmol) in anhydrous MeOH (0.33 mL), Et_3_N (14 μL, 0.1 mmol) and boc anhydride (23 mg, 0.1 mmol) were added and the mixture was stirred at room temperature for 4 h. The resulting solution was concentrated to dryness and the oily residue was diluted in DCM and washed successively with ice-cooled citric acid_(aq)_ 5%, water and brine, two times. The organic layer was then dried over anhydrous Na_2_SO_4_, filtered and concentrated under vacuum. The desired product was afforded in pure form as a colorless oil (32 mg, 82% yield) after FCC purification of the residue using ethyl acetate as eluent. R*f* (EtOAc/CH_3_COOH) 10:0.02 = 0.45; ^1^H NMR (CDCl_3_, 600.13 MHz) δ 7.07 (d, *J* = 7.8 Hz, 2 H), 6.62 (d, *J* = 9 Hz, 2 H), 4.94 (d, *J* = 7.8 Hz, 1 H), 4.54 (d, *J* = 9 Hz, 1 H), 3.71 (t, *J* = 7.2 Hz, 4 H), 3.61 (t, *J* = 7.2 Hz, 4 H), 3.09 (dd, *J* = 5.4 Hz, *J*′ = 14.4 Hz, 1 H), 3.00 (dd, *J* = 6 Hz, *J*′ = 15 Hz, 1 H), 1.43 (s, 9 H); ^13^C NMR (CDCl_3_, 150.9 MHz) δ 176.3, 155.5, 145.2, 130.7, 124.6, 112.1, 80.3, 60.4, 54.4, 53.5, 40.4, 36.7, 30.9, 28.3.

*Tert*-butyl((*S*)-3-(4-(bis(2-chloroethyl)amino)phenyl)-1-(((*S*)-3-methyl-1-((3a*S*,4*S*,6*S*,7a*R*)-3a,5,5-trimethylhexahydro-4,6-methanobenzo[*d*][1,3,2]dioxaborol-2-yl)butyl)amino)-1-oxopropan-2-yl)carbamate (**E4**): To a stirring solution of *N*′-Boc-melphalan (180 mg, 0.44 mmol) in anhydrous DMF (0.88 mL), DIPEA (300 μL, 1.76 mmol) and HATU (186 mg, 0.49 mmol) were added and the mixture was cooled at 0 °C for 15 min. Then, (*R*)-boroleucine (1*S*,2*S*,3*R*,5*S*)-(+)-2,3-pinanediol ester trifluoroacetate salt (167 mg, 0.44 mmol) was added and the reaction mixture was left under stirring at 0 °C for 30 min and subsequently at room temperature for 20 h. Upon completion, the solution was diluted in DCM and washed with ice-cooled water and brine, twice. The organic layer was then dried over anhydrous Na_2_SO_4_, filtered and concentrated under vacuum. The pure product was afforded as a colorless oil (200 mg, 70% yield) after FCC purification of the oily residue, using toluene/ethyl acetate 99:1 as eluent. R*f* (PhMe/EtOAc) 7:3 = 0.5; ^1^H NMR (CDCl_3_, 600.13 MHz) δ 7.10 (d, *J* = 7.8 Hz, 2 H), 6.60 (d, *J* = 8.4 Hz, 2 H), 4.30 (dd, *J* = 1.8 Hz, *J*′ = 8.4 Hz, 2 H), 3.70 (t, *J* = 7.2 Hz, 4 H), 3.61 (t, *J* = 7.2 Hz, 4 H), 3.07 (td, *J* = 4.2 Hz, *J*′ = 4.8 Hz, 1 H), 3.00 (dd, *J* = 6 Hz, *J*′ = 13.8 Hz, 1 H), 2.93 (dd, *J* = 7.2 Hz, *J*′ = 14.4 Hz, 1 H), 2.34–2.36 (m, 1 H), 2.32–2.33 (m, 1 H), 2.23–2.25 (m, 2 H), 2.00–2.05 (m, 3 H), 1.90–1.93 (m, 2 H), 1.88–1.90 (m, 1 H), 1.85–1.86 (m, 2 H), 1.41 (s, 9 H), 1.28 (s, 6 H), 0.85–0.87 (m, 6 H), 0.83 (s, 3 H); ^13^C NMR (CDCl_3_, 150.9 MHz) δ 167.7, 144.4, 140.4, 130.8, 129.1, 129.0, 118.3, 116.0, 112.2, 85.4, 84.7, 69.3, 53.5, 51.5, 40.4, 39.7, 39.4, 38.4, 38.2, 37.1, 35.7, 28.7, 28.6, 28.3, 27.2, 27.1, 26.3, 25.4, 24.1, 24.0, 23.1, 21.9, 13.7.

(*S*)-3-(4-(bis(2-chloroethyl)amino)phenyl)-1-(((*S*)-3-methyl-1-((3a*S*,4*S*,6*S*,7a*R*)-3a,5,5-trimethylhexahydro-4,6-methanobenzo[*d*][1,3,2]dioxaborol-2-yl)butyl)amino)-1-oxopropan-2-aminium chloride salt (**E6**): Trifluoroethanol (9 μL, 0.12 mmol) and 4 M HCl in dioxane (35 μL, 0.14 mmol) were dripped into an ice-cooled solution of *tert*-butyl ((*S*)-3-(4-(bis(2-chloroethyl)amino)phenyl)-1-(((*S*)-3-methyl-1-((3a*S*,4*S*,6*S*,7a*R*)-3a,5,5-trimethylhexahydro-4,6-methanobenzo[*d*][1,3,2]dioxaborol-2-yl)butyl)amino)-1-oxopropan-2-yl)carbamate (77 mg, 0.12 mmol) in anhydrous DCM (0.3 mL) and the resulting mixture was initially stirred at 0 °C and then at room temperature for 2 h. Addition of 4 M HCl in dioxane took place, if needed. Upon consumption of the starting material, concentration of the solution took place, followed by successive dropwise addition of diethyl ether and hexane, respectively. The white precipitate was washed thrice with hexane, and the desired salt was afforded as white amorphous solid (67 mg, 96% yield).

(2*S*)-*N*-((1*R*)-3-methyl-1-((3a*S*,4*S*,6*S*)-3a,5,5-trimethylhexahydro-4,6-methanobenzo[*d*][1,3,2]dioxaborol-2-yl)butyl)-3-phenyl-2-(tritylamino)propenamide (**E3**): To a stirring solution of *N*′-trityl-*L*-phenylalanine (389 mg, 0.93 mmol) in anhydrous DMF (1.85 mL), DIPEA (500 μL, 2.77 mmol) and HATU (389 mg, 1.02 mmol) were added and the mixture was cooled at 0 °C for 15 min. Then, (*R*)-boroleucine (1*S*,2*S*,3*R*,5*S*)-(+)-2,3-pinanediol ester trifluoroacetate salt (350 mg, 0.92 mmol) was added and the reaction mixture was left under stirring at 0 °C for 30 min and subsequently at room temperature for 20 h. Upon completion, the solution was diluted in DCM and washed with ice-cooled water and brine, twice. The organic layer was then dried over anhydrous Na_2_SO_4_, filtered and concentrated under vacuum. The pure product was afforded as a white foam (410 mg, 68% yield) after FCC purification of the oily residue, using toluene/ethyl acetate 99:1 as eluent. R*f* (PhMe/EtOAc) 95:5 = 0.24; ^1^H NMR (CDCl_3_, 600.13 MHz) δ 7.43 (d, *J* = 7.8 Hz, 7 H), 7.25–7.29 (m, 4 H), 7.20–7.22 (m, 7 H), 6.92 (d, *J* = 6.6 Hz, 2 H), 4.29 (d, *J* = 8.4 Hz, 1 H), 3.57 (t, *J* = 6 Hz, 1 H), 3.00–3.02 (m, 1 H), 2.96 (dd, *J* = 3.6 Hz, *J*′ = 13.2 Hz, 1 H), 2.33–2.35 (m, 1 H), 2.24–2.26 (m, 1 H), 2.16–2.18 (m, 1 H), 2.04 (dd, *J* = 6 Hz, *J*′ = 12 Hz, 2 H), 1.86–1.90 (m, 2 H), 1.61–1.63 (m, 3 H), 1.51–1.54 (hept, *J* = 6.6 Hz, 1 H), 1.42 (s, 3 H), 1.27 (s, 3 H), 0.95–0.96 (m, 1 H), 0.90 (d, *J* = 6.6 Hz, 3 H), 0.88 (d, *J* = 6.6 Hz, 3 H), 0.85 (s, 3 H); ^13^C NMR (CDCl_3_, 150.9 MHz) δ 175.7, 145.9, 136.0, 130.2, 128.9, 128.7, 128.6, 128.5, 128.2, 128.0, 126.8, 126.7, 84.9, 77.4, 72.0, 64.4, 57.0, 51.7, 40.5, 39.8, 38.1, 37.8, 36.0, 30.6, 28.9, 27.2, 26.5, 25.8, 24.1, 23.0, 22.3.

(2*S*)-1-(((1*R*)-3-methyl-1-((3a*S*,4*S*,6*S*)-3a,5,5-trimethylhexahydro-4,6-methanobenzo[*d*][1,3,2]dioxaborol-2-yl)butyl)amino)-1-oxo-3-phenylpropan-2-aminium chloride salt (**E5**): Trifluoroethanol (104 μL, 1.45 mmol) and 4 M HCl in dioxane (815 μL, 3.25 mmol) were dripped into an ice-cooled solution of (2*S*)-*N*-((1*R*)-3-methyl-1-((3a*S*,4*S*,6*S*)-3a,5,5-trimethylhexahydro-4,6-methanobenzo[*d*][1,3,2]dioxaborol-2-yl)butyl)-3-phenyl-2-(tritylamino)propenamide (950 mg, 1.45 mmol) in anhydrous DCM (14.5 mL) and the resulting mixture was initially stirred at 0 °C and then at room temperature for 2 h. Upon consumption of the starting material, concentration of the solution up to 1 mL took place, followed by successive dropwise addition of diethyl ether and hexane, respectively. The white precipitate was washed with hexane thrice, and the desired salt was afforded as white amorphous solid (636 mg, 98% yield).

*N*^1^-(2-aminophenyl)-*N*^4^-((2*R*)-1-(((1*R*)-3-methyl-1-((3a*S*)-3a,5,5-trimethylhexahydro-4,6-methanobenzo[*d*][1,3,2]dioxaborol-2-yl)butyl)amino)-1-oxo-3-phenylpropan-2-yl)terephthalamide (**2**): To a stirring solution of 4-((2-aminophenyl) carbamoyl) benzoic acid (50 mg, 0.2 mmol) in a mixture of anhydrous DCM (133 μL) and DMF (260 μL), *N*′-methyl morpholine (90 μL, 0.8 mmol) and TBTU (83 mg, 0.26 mmol) were added and the mixture was cooled at 0 °C. Then, (2*S*)-1-(((1*R*)-3-methyl-1-((3a*S*,4*S*,6*S*)-3a,5,5-trimethylhexahydro-4,6-methanobenzo[*d*][1,3,2]dioxaborol-2-yl)butyl)amino)-1-oxo-3-phenylpropan-2-aminium chloride salt (89 mg, 0.2 mmol) was added and the reaction mixture was left under stirring at 0 °C for 30 min and subsequently at room temperature for 20 h. Upon completion, the mixture was subjected to FCC purification using toluene/ethyl acetate 8:2 as eluent, and the hybrid was afforded as pale-yellow solid (70 mg, 53% yield). R*f* (PhMe/EtOAc) 8:2 = 0.15; IR (KBr): 2962, 1519, 1120, 1020 cm^−1^; HR-MALDI (*m*/*z*) [M+Νa^+^] calculated for [C_38_H_47_BN_4_NaO_5_]^+^ 673.3532. Found: 673.1960; ^1^H NMR (CDCl_3_, 600.13 MHz) δ 8.40 (brs, 1 H), 7.90 (s, 2 H), 7.72 (s, 2 H), 7.37 (s, 1 H), 7.11 (s, 2 H), 6.90 (s, 2 H), 6.17 (brs, 1 H), 4.80 (s, 1 H), 4.31 (d, *J* = 8.4 Hz, 1 H), 3.35–3.36 (m, 1 H), 3.13–3.15 (m, 3 H), 2.35–2.37 (m, 2 H), 2.19–2.20 (m, 1 H), 2.00–2.04 (hept, *J* = 5.4 Hz, 1 H), 1.87–1.89 (m, 3 H), 1.37–1.39 (m, 5 H), 1.28–1.30 (m, 8 H), 0.81–0.84 (m, 10 H); ^13^C NMR (CDCl_3_, 150.9 MHz) δ ^13^C NMR (151 MHz, CDCl_3_) δ 129.5, 128.6, 127.9, 127.7, 127.4, 127.0, 69.3, 54.5, 54.0, 51.4, 40.5, 40.0, 39.6, 39.0, 38.6, 38.2, 35.6, 29.7, 29.6, 28.7, 28.0, 27.8, 27.1, 26.4, 25.3, 24.1, 24.0, 23.0, 21.9.

*N*^2^-(2-aminophenyl)-*N*^5^-((2*R*)-1-(((1*R*)-3-methyl-1-((3a*S*)-3a,5,5-trimethylhexahydro-4,6-methanobenzo[*d*][1,3,2]dioxaborol-2-yl)butyl)amino)-1-oxo-3-phenylpropan-2-yl)pyrazine-2,5-dicarboxamide (**3**): To a stirring solution of 5-((2-aminophenyl)carbamoyl)pyrazine-2-carboxylic acid (20 mg, 0.08 mmol) in a mixture of anhydrous DCM (95 μL) and DMF (103 μL), *N*′-methyl morpholine (50 μL, 0.23 mmol) and TBTU (28 mg, 0.09 mmol) were added and the mixture was cooled at 0 °C. Then, (2*S*)-1-(((1*R*)-3-methyl-1-((3a*S*,4*S*,6*S*)-3a,5,5-trimethylhexahydro-4,6-methanobenzo[*d*][1,3,2]dioxaborol-2-yl)butyl)amino)-1-oxo-3-phenylpropan-2-aminium chloride salt (35 mg, 0.08 mmol) was added and the reaction mixture was left under stirring at 0 °C for 30 min and subsequently at room temperature for 20 h. Upon completion, the mixture was subjected to FCC purification using toluene/ethyl acetate 7:3 as eluent, and the hybrid was afforded as a bright yellow oil (25 mg, 50% yield). R*f* (PhMe/EtOAc) 7:3 = 0.11; IR (KBr): 2962, 1652, 1519, 1020 cm^−1^; HR-MALDI (*m*/*z*) [M+Νa^+^] calculated for [C_36_H_45_BN_6_NaO_5_]^+^ 675.3437. Found: 675.0880; ^1^H NMR (CDCl_3_, 600.13 MHz) δ 9.59 (s, 1 H), 9.40 (d, *J* = 1.2 Hz, 1 H), 9.31 (d, *J* = 1.2 Hz, 1 H), 8.46 (d, *J* = 8.4 Hz, 1 H), 7.52 (dd, *J* = 1.2 Hz, *J*′ = 7.8 Hz, 1 H), 7.28–7.30 (m, 3 H), 7.23 (tt, *J* = 2.4 Hz, *J*′ = 5.4 Hz, 1 H), 7.12 (td, *J* = 1.8 Hz, *J*′ = 7.8 Hz, 1 H), 6.87–6.89 (m, 2 H), 5.90 (d, *J* = 5.4 Hz, 1 H), 4.80 (td, *J* = 6.6 Hz, *J*′ = 8.4 Hz, 1 H), 4.32 (dd, *J* = 2.4 Hz, *J*′ = 9 Hz, 1 H), 3.86–3.88 (m, 2 H), 3.17–3.19 (m, 3 H), 2.34 (ddt, *J* = 3 Hz, *J*′ = 8.4 Hz, *J*″ = 14.4 Hz, 1 H), 2.23–2.25 (m, 3 H), 2.00–2.02 (m, 1 H), 1.91–1.93 (m, 1 H), 1.84 (ddd, *J* = 1.2 Hz, *J*′ = 2.4 Hz, *J*″ = 14.4 Hz, 1 H), 1.38–1.40 (m, 5 H), 1.27–1.29 (m, 4 H), 0.85 (d, *J* = 4.8 Hz, 6 H), 0.83 (d, *J* = 3.6 Hz, 3 H); ^13^C NMR (150.9 MHz, CDCl_3_) δ 170.2, 162.0, 160.2, 146.1, 142.6, 142.1, 140.1, 136.3, 129.4, 128.7, 127.4, 127.1, 124.5, 123.7, 119.9, 118.4, 86.0, 79.9, 54.4, 54.0, 51.4, 39.9, 39.6, 38.6, 38.2, 35.5, 29.7, 28.5, 28.0, 27.8, 27.1, 26.3, 25.3, 24.0, 23.0, 22.0.

*N*^1^-(4-((2-aminophenyl)carbamoyl)benzyl)-*N*^4^-((2*R*)-1-(((1*R*)-3-methyl-1-((3a*R*)-3a,5,5-trimethylhexahydro-4,6-methanobenzo[*d*][1,3,2]dioxaborol-2-yl)butyl)amino)-1-oxo-3-phenylpropan-2-yl)terephthalamide (**4**): To a stirring solution of 4-((4-((2-aminophenyl)carbamoyl)benzyl)carbamoyl)benzoic acid (42 mg, 0.11 mmol) in a mixture of anhydrous DCM (73 μL) and DMF (147 μL), *N*′-methyl morpholine (50 μL, 0.44 mmol) and TBTU (50 mg, 0.15 mmol) were added and the mixture was cooled at 0 °C. Then, (2*S*)-1-(((1*R*)-3-methyl-1-((3a*S*,4*S*,6*S*)-3a,5,5-trimethylhexahydro-4,6-methanobenzo[*d*][1,3,2]dioxaborol-2-yl)butyl)amino)-1-oxo-3-phenylpropan-2-aminium chloride salt (50 mg, 0.11 mmol) was added and the reaction mixture was left under stirring at 0 °C for 30 min and subsequently at room temperature for 20 h. Upon completion, the mixture was subjected to FCC purification using toluene/ethyl acetate 8:2 as eluent, and the hybrid was afforded as a colorless oil (39 mg, 45% yield). R*f* (PhMe/EtOAc) 8:2 = 0.12; IR (KBr): 2962, 1544, 1120, 1020 cm^−1^; HR-MALDI (*m*/*z*) [M+Νa^+^] calculated for [C_46_H_54_BN_5_NaO_6_]^+^ 806.4059. Found: 806.1370; ^1^H NMR (CDCl_3_, 600.13 MHz) δ 9.58 (brs, 1 H), 9.10 (brs, 1 H), 8.01 (brs, 1 H), 7.95 (d, *J* = 7.8 Hz, 1 H), 7.81 (brs, 1 H), 7.56 (d, *J* = 7.2 Hz, 4 H), 7.51 (brs, 1 H), 7.41 (brs, 1 H), 7.28–7.32 (m, 5 H), 7.19–7.21 (m, 3 H), 7.09 (brs, 1 H), 7.02 (brs, 1 H), 5.87 (s, 1 H), 4.79 (d, *J* = 7.2 Hz, 1 H), 4.30–4.31 (m, 1 H), 3.42 (s, 1 H), 3.19–3.20 (m, 1 H), 3.11–3.12 (m, 1 H), 2.19–2.20 (m, 1 H), 2.02–2.03 (m, 1 H), 1.91–1.92 (m, 1 H), 1.85 (d, *J* = 14.4 Hz, 1 H), 1.60 (s, 4 H), 1.39 (m, 4 H), 1.28–1.29 (m, 6 H), 0.83–0.85 (m, 7 H); ^13^C NMR (150.9 MHz, CDCl_3_) δ 145.8, 136.5, 129.5, 129.0, 128.7, 128.6, 128.2, 128.1, 128.0, 127.4, 126.5, 86.0, 77.9, 54.0, 51.3, 40.0, 39.5, 38.7, 38.2, 35.5, 28.6, 27.1, 26.3, 25.3, 24.0, 23.0, 22.0.

*N*^2^-(4-((2-aminophenyl)carbamoyl)benzyl)-*N*^5^-((2*R*)-1-(((1*R*)-3-methyl-1-((3a*R*)-3a,5,5-trimethylhexahydro-4,6-methanobenzo[*d*][1,3,2]dioxaborol-2-yl)butyl)amino)-1-oxo-3-phenylpropan-2-yl)pyrazine-2,5-dicarboxamide (**5**): To a stirring solution of 5-((4-((2-aminophenyl)carbamoyl)benzyl)carbamoyl)pyrazine-2-carboxylic acid (30 mg, 0.08 mmol) in a mixture of anhydrous DCM (51 μL) and DMF (100 μL), *N*′-methyl morpholine (30 μL, 0.23 mmol) and TBTU (30 mg, 0.09 mmol) were added and the mixture was cooled at 0 °C. Then, (2*S*)-1-(((1*R*)-3-methyl-1-((3a*S*,4*S*,6*S*)-3a,5,5-trimethylhexahydro-4,6-methanobenzo[*d*][1,3,2]dioxaborol-2-yl)butyl)amino)-1-oxo-3-phenylpropan-2-aminium chloride salt (35 mg, 0.08 mmol) was added and the reaction mixture was left under stirring at 0 °C for 30 min and subsequently at room temperature for 20 h. Upon completion, the mixture was subjected to FCC purification using toluene/ethyl acetate 8:2 as eluent, and the hybrid was afforded as a yellow oil (24 mg, 40% yield). R*f* (PhMe/EtOAc) 8:2 = 0.29; IR (KBr): 2962, 1729, 1616, 1120, 1020 cm^−1^; HR-MALDI (*m*/*z*) [M+Νa^+^] calculated for [C_44_H_52_BN_7_NaO_6_]^+^ 808.3964. Found: 808.2430; ^1^H NMR (CDCl_3_, 600.13 MHz) δ 10.05 (s, 1 H), 9.39 (s, 1 H), 9.26 (s, 1 H), 9.12 (s, 1 H), 8.43 (d, *J* = 8.4 Hz, 1 H), 7.93 (d, *J* = 7.8 Hz, 1 H), 7.76 (d, *J* = 7.8 Hz, 1 H), 7.60 (d, *J* = 7.8 Hz, 1 H), 7.39 (d, *J* = 7.8 Hz, 2 H), 7.28 (d, *J* = 4.8 Hz, 2 H), 5.90 (s, 1 H), 5.00 (s, 1 H), 4.78 (q, *J* = 7.2 Hz, 1 H), 4.32–4.34 (m, 2 H), 3.98 (dd, *J* = 4.8 Hz, *J*′ = 9 Hz, 1 H), 3.15–3.17 (m, 2 H), 2.55 (s, 1 H), 2.44–2.45 (m, 1 H), 2.33–2.34 (m, 1 H), 2.19–2.20 (m, 2 H), 2.01 (q, *J* = 5.4 Hz, 2 H), 1.92 (dt, *J* = 2.4 Hz, *J*′ = 6.6 Hz, 2 H), 1.82–1.83 (m, 1 H), 1.63 (ddd, *J* = 2.4 Hz, *J*′ = 5.4 Hz, *J*″ = 13.8 Hz, 1 H), 1.46 (s, 4 H), 1.37–1.39 (m, 3 H), 1.26–1.28 (m, 6 H), 0.82–0.85 (m, 9 H); ^13^C NMR (150.9 MHz, CDCl_3_) δ 165.7, 142.2, 129.4, 128.7, 127.8, 127.0, 126.5, 77.9, 54.0, 51.3, 40.5, 39.9, 39.5, 38.2, 35.5, 29.6, 28.5, 28.4, 28.0, 27.8, 27.1, 26.3, 25.3, 24.1, 24.0, 22.9, 22.0.

*N*^1^-(4-((2-aminophenyl)carbamoyl)benzyl)-*N*^4^-((2*R*)-1-(((1*R*)-3-methyl-1-((3a*R*)-3a,5,5-trimethylhexahydro-4,6-methanobenzo[*d*][1,3,2]dioxaborol-2-yl)butyl)amino)-1-oxo-3-phenylpropan-2-yl)succinimide (**1**): To a stirring solution of 4-((4-((2-aminophenyl) carbamoyl)benzyl)amino)-4-oxobutanoic acid (20 mg, 0.06 mmol) in a mixture of anhydrous DCM (40 μL) and DMF (80 μL), DIPEA (42 μL, 0.24 mmol) and pyBOP (52 mg, 0.1 mmol) were added and the mixture was cooled at 0 °C. Then, (2*S*)-1-(((1*R*)-3-methyl-1-((3a*S*,4*S*,6*S*)-3a,5,5-trimethylhexahydro-4,6-methanobenzo[*d*][1,3,2]dioxaborol-2-yl)butyl)amino)-1-oxo-3-phenylpropan-2-aminium chloride salt (27 mg, 0.06 mmol) was added and the reaction mixture was left under stirring at 0 °C for 30 min and subsequently at room temperature for 20 h. Upon completion, the mixture was subjected to FCC purification using ethyl acetate/methanol 9:1 as eluent, and the hybrid was afforded as a white solid (crystallization in ethyl acetate) (26 mg, 60% yield). R*f* (EtOAc/MeOH) 9:1 = 0.09; IR (KBr): 1519, 1120, 1020 cm^−1^; HR-ESI (*m*/*z*) [M+H^+^] calculated for [C_42_H_55_BN_5_O_6_]^+^ 736.4240 Found: 736.4239; ^1^H NMR (CDCl_3_, 600.13 MHz) δ 8.41 (s, 1 H), 7.75 (d, *J* = 7.2 Hz, 2 H), 7.45–7.46 (m, 1 H), 7.30–7.33 (m, 3 H), 7.18–7.22 (m, 3 H), 6.80 (s, 1 H), 6.63 (s, 1 H), 6.51 (s, 1 H), 4.66 (q, *J* = 7.2 Hz, 1 H), 4.48 (dd, *J* = 6 Hz, *J*′ = 15.6 Hz, 1 H), 4.39 (dd, *J* = 5.4 Hz, *J*′ = 15 Hz, 1 H), 4.29 (dd, *J* = 1.8 Hz, *J*′ = 8.4 Hz, 1 H), 3.06 (qd, *J* = 6 Hz, *J*′ = 13.8 Hz, 2 H), 2.90–2.91 (m, 1 H), 2.85 (s, 3 H), 2.59–2.60 (m, 1 H), 2.44–2.48 (m, 3 H), 2.32–2.33 (m, 1 H), 2.17 (ddt, *J* = 1.8 Hz, *J*′ = 6 Hz, *J*″ = 7.8 Hz, 1 H), 2.02–2.04 (m, 2 H), 1.90–1.91 (m, 1 H), 1.82 (dt, *J* = 3 Hz, *J*′ = 14.4 Hz, 1 H), 1.75 (s, 3 H), 1.48 (dq, *J* = 6.6 Hz, *J*′ = 13.2 Hz, 1 H), 1.40 (s, 3 H), 1.34 (t, *J* = 7.2 Hz, 2 H), 1.28 (s, 3 H), 0.85 (s, 3 H), 0.80 (d, *J* = 6.6 Hz, 3 H), 0.76 (d, *J* = 6.6 Hz, 3 H); ^13^C NMR (150.9 MHz, CDCl_3_) δ 176.4, 172.3, 171.8, 129.6, 129.4, 128.6, 128.4, 127.7, 127.0, 85.3, 53.1, 51.6, 40.0, 39.7, 38.2, 35.8, 28.7, 27.2, 26.4, 25.2, 24.1, 23.0, 22.0.

*N*-(2-aminophenyl)-4-(2-(((2*R*)-1-(((1*R*)-3-methyl-1-((3a*R*)-3a,5,5-trimethylhexahydro-4,6-methanobenzo[*d*][1,3,2]dioxaborol-2-yl)butyl)amino)-1-oxo-3-phenylpropan-2-yl)amino)-2-oxoethyl)benzamide (**6**): To a stirring solution of 2-(4-((2-aminophenyl)carbamoyl)phenyl) acetic acid (31 mg, 0.12 mmol) in a mixture of anhydrous DCM (106 μL) and DMF (153 μL), *N*′-methyl morpholine (40 μL, 0.35 mmol) and TBTU (41 mg, 0.13 mmol) were added and the mixture was cooled at 0 °C. Then, (2*S*)-1-(((1*R*)-3-methyl-1-((3a*S*,4*S*,6*S*)-3a,5,5-trimethylhexahydro-4,6-methanobenzo[*d*][1,3,2]dioxaborol-2-yl)butyl)amino)-1-oxo-3-phenylpropan-2-aminium chloride salt (51 mg, 0.12 mmol) was added and the reaction mixture was left under stirring at 0 °C for 30 min and subsequently at room temperature for 20 h. Upon completion, the mixture was subjected to FCC purification using toluene/ethyl acetate 8:2 as eluent, and the hybrid was afforded as a colorless oil (47 mg, 62% yield). R*f* (PhMe/EtOAc) 1:1 = 0.33; IR (KBr): 2925, 1662, 1533, 1027 cm^−1^; HR-ESI (*m*/*z*) [M+Na^+^] calculated for [C_39_H_49_BN_4_NaO_5_]^+^ 687.3688 Found: 687.3690; ^1^H NMR (CDCl_3_, 600.13 MHz) δ 8.25 (s, 1 H), 7.82 (d, *J* = 7.8 Hz, 2 H), 7.31 (d, *J* = 7.8 Hz, 1 H), 7.20–7.24 (m, 5 H), 7.07–7.13 (m, 3 H), 6.84 (dd, *J* = 8.4 Hz, *J*′ = 7.8 Hz, 2 H), 6.51 (d, *J* = 7.8 Hz, 1 H), 6.14 (d, *J* = 5.4 Hz, 1 H), 4.60 (q, *J* = 7.2 Hz, 1 H), 4.29 (dd, *J* = 2.4 Hz, *J*′ = 9 Hz, 1 H), 3.53 (s, 2 H), 3.10 (dt, *J* = 6 Hz, *J*′ = 9 Hz, 1 H), 2.96 (dd, *J* = 1.8 Hz, *J*′ = 6.6 Hz, 2 H), 2.32–2.34 (m, 1 H), 2.17-2.19 (m, 1 H), 2.02 (dt, *J* = 5.4 Hz, *J*′ = 10.8 Hz, 1 H), 1.89–1.91 (m, 3 H), 1.37–1.39 (m, 6 H), 1.27–1.29 (m, 6 H), 0.81–1.85 (m, 9 H); ^13^C NMR (150.9 MHz, CDCl_3_) δ 170.9, 170.0, 165.6, 140.8, 138.6, 136.3, 133.0, 129.6, 129.4, 128.5, 127.9, 127.3, 126.9, 124.6, 119.7, 118.3, 85.8, 77.8, 73.8, 69.2, 53.8, 51.4, 43.2, 40.5, 40.0, 39.5, 38.3, 38.2, 35.6, 29.6, 28.6, 28.0, 27.8, 27.1, 26.3, 25.3, 24.0, 23.0, 22.0.

*N*-(2-amino-4-fluorophenyl)-4-(2-(((2*R*)-1-(((1*R*)-3-methyl-1-((3a*R*)-3a,5,5-trimethyl-hexahydro-4,6-methanobenzo[*d*][1,3,2]dioxaborol-2-yl)butyl)amino)-1-oxo-3-phenylpropan-2-yl)amino)-2-oxoethyl)benzamide (**7**): To a stirring solution of 2-(4-((2-amino-4-fluorophenyl)carbamoyl)phenyl) acetic acid (37 mg, 0.13 mmol) in a mixture of anhydrous DCM (85 μL) and DMF (170 μL), *N*′-methyl morpholine (43 μL, 0.39 mmol) and TBTU (46 mg, 0.14 mmol) were added and the mixture was cooled at 0 °C. Then, (2*S*)-1-(((1*R*)-3-methyl-1-((3a*S*,4*S*,6*S*)-3a,5,5-trimethylhexahydro-4,6-methanobenzo[*d*][1,3,2]dioxaborol-2-yl)butyl)amino)-1-oxo-3-phenylpropan-2-aminium chloride salt (58 mg, 0.13 mmol) was added and the reaction mixture was left under stirring at 0 °C for 30 min and subsequently at room temperature for 20 h. Upon completion, the mixture was subjected to FCC purification using toluene/ethyl acetate 6:4 as eluent, and the hybrid was afforded as a colorless oil (56 mg, 63% yield). R*f* (PhMe/EtOAc) 6:4 = 0.1; IR (KBr): 2925, 1662, 1533, 1027 cm^−1^; HR-ESI (*m*/*z*) [M+Na^+^] calculated for [C_39_H_48_BFN_4_NaO_5_]^+^ 705.3594 Found: 705.3599; ^1^H NMR (CDCl_3_, 600.13 MHz) δ 8.17 (s, 1 H), 7.81 (d, *J* = 7.8 Hz, 2 H), 7.20–7.24 (m, 5 H), 7.15 (dd, *J* = 6 Hz, *J*′ = 8.4 Hz, 1 H), 7.10–7.12 (m, 2 H), 6.56 (d, *J* = 7.8 Hz, 1 H), 6.50 (tt, *J* = 2.4 Hz, *J*′ = 9.6 Hz, 2 H), 6.11 (d, *J* = 5.4 Hz, 1 H), 4.59 (q, *J* = 7.2 Hz, 1 H), 4.28 (dd, *J* = 1.8 Hz, *J*′ = 8.4 Hz, 1 H), 3.52 (s, 2 H), 3.11 (dt, *J* = 1.2 Hz, *J*′ = 6 Hz, 1 H), 2.95–2.97 (m, 2 H), 2.33 (ddq, *J* = 2.4 Hz, *J*′ = 9 Hz, 1 H), 2.18–2.19 (m, 1 H), 2.02 (t, *J* = 5.4 Hz, 1 H), 1.90 (tt, *J* = 3 Hz, *J*′ = 6 Hz, 1 H), 1.82 (dt, *J* = 2.4 Hz, *J*′ = 14.4 Hz, 1 H), 1.43–1.45 (m, 1 H), 1.39 (s, 4 H), 1.34–1.35 (m, 2 H), 1.28 (s, 4 H), 1.19 (d, *J* = 10.8 Hz, 1 H), 0.83 (m, 9 H); ^13^C NMR (150.9 MHz, CDCl_3_) δ 170.9, 169.9, 165.9, 162.7, 161.1, 143.4, 138.8, 136.3, 132.7, 129.5, 129.4, 128.5, 127.9, 126.9, 119.8, 105.8, 105.7, 104.3, 104.1, 85.9, 77.8, 69.2, 53.9, 51.4, 43.1, 40.0, 39.5, 38.6, 38.4, 38.2, 35.5, 28.6, 27.1, 26.3, 25.3, 24.0, 22.9, 22.0.

*N*^2^-(2-aminophenyl)-*N*^5^-((1*R*)-3-methyl-1-((3a*S*)-3a,5,5-trimethylhexahydro-4,6-methanobenzo[*d*][1,3,2]dioxaborol-2-yl)butyl)pyrazine-2,5-dicarboxamide (**10**): To a stirring solution of 5-((2-aminophenyl)carbamoyl)pyrazine-2-carboxylic acid (20 mg, 0.1 mmol) in a mixture of anhydrous DCM (52 μL) and DMF (104 μL), *N*′-methyl morpholine (30 μL, 24 mmol) and TBTU (28 mg, 0.1 mmol) were added and the mixture was cooled at 0 °C. Then, (*R*)-boroleucine (1*S*,2*S*,3*R*,5*S*)-(+)-2,3-pinanediol ester trifluoroacetate salt (30 mg, 0.1 mmol) was added and the reaction mixture was left under stirring at 0 °C for 30 min and subsequently at room temperature for 20 h. Upon completion, the mixture was subjected to FCC purification using toluene/ethyl acetate 9:1 as eluent, and the hybrid was afforded as a bright yellow oil (16 mg, 40% yield). R*f* (PhMe/EtOAc) 9:1 = 0.27; IR (KBr): 2962, 1652, 1519, 1020, 802 cm^−1^; HR-ESI (*m*/*z*) [M+H^+^] calculated for [C_27_H_40_BN_5_O_4_]^+^ 506.2933 Found: 506.2938; ^1^H NMR (CDCl_3_, 600.13 MHz) δ 9.60 (s, 1 H), 9.41 (d, *J* = 1.8 Hz, 1 H), 9.36 (d, *J* = 1.8 Hz, 1 H), 7.94 (d, *J* = 6.6 Hz, 1 H), 7.52 (dd, *J* = 1.8 Hz, *J* = 7.8 Hz, 1 H), 7.11 (td, *J* = 1.2 Hz, *J*′ = 7.8 Hz, 1 H), 6.87–6.89 (m, 2 H), 4.37 (dd, *J* = 2.4 Hz, *J*′ = 9 Hz, 1 H), 3.63 (dt, *J* = 6 Hz, *J*′ = 9.6 Hz, 1 H), 2.35–2.37 (m, 1 H), 2.22–2.24 (m, 1 H), 2.07 (t, *J* = 5.4 Hz, 1 H), 1.91–1.93 (m, 3 H), 1.75 (ddt, *J* = 1.8 Hz, *J*′ = 6.6 Hz, 1 H), 1.66–1.68 (m, 1 H), 1.60–1.61 (m, 1 H), 1.45 (s, 3 H), 1.28–1.30 (m, 5 H), 0.96–1.00 (m, 6 H), 0.85 (s, 3 H); ^13^C NMR (150.9 MHz, CDCl_3_) δ 162.2, 146.6, 145.8, 142.4, 142.2, 127.3, 124.5, 123.8, 119.9, 118.3, 86.5, 78.2, 51.3, 40.2, 39.5, 38.2, 35.4, 28.5, 27.1, 26.4, 25.6, 24.0, 23.1, 22.0.

*N*^2^-(2-aminophenyl)-*N*^5^-((2*R*)-3-(4-(bis(2-chloroethyl)amino)phenyl)-1-(((1*R*)-3-methyl-1-((3a*S*)-3a,5,5-trimethylhexahydro-4,6-methanobenzo[*d*][1,3,2]dioxaborol-2-yl)butyl)amino)-1-oxopropan-2-yl)pyrazine-2,5-dicarboxamide (**11**): To a stirring solution of 5-((2-aminophenyl)carbamoyl)pyrazine-2-carboxylic acid (10 mg, 0.04 mmol) in a mixture of anhydrous DCM (20 μL) and DMF (60 μL), *N*′-methyl morpholine (20 μL, 0.12 mmol) and TBTU (20 mg, 0.05 mmol) were added and the mixture was cooled at 0 °C. Then, (*S*)-3-(4-(bis(2-chloroethyl)amino)phenyl)-1-(((*S*)-3-methyl-1-((3a*S*,4*S*,6*S*,7a*R*)-3a,5,5-trimethylhexahydro-4,6-methanobenzo[*d*][1,3,2]dioxaborol-2-yl)butyl)amino)-1-oxopropan-2-aminium chloride salt (25 mg, 0.04 mmol) was added and the reaction mixture was left under stirring at 0 °C for 30 min and subsequently at room temperature for 20 h. Upon completion, the mixture was subjected to FCC purification using toluene/ethyl acetate 6:4 as eluent, and the hybrid was afforded as a bright yellow oil (13 mg, 41% yield). R*f* (PhMe/EtOAc) 1:1 = 0.18; IR (KBr): 2962, 1652, 1519, 1020, 802 cm^−1^; HR-ESI (*m*/*z*) [M+H^+^] calculated for [C_40_H_53_BCl_2_N_7_O_5_]^+^ 792.3573 Found: 792.3594; ^1^H NMR (CDCl_3_, 600.13 MHz) δ 9.60 (s, 1 H), 9.42 (d, *J* = 1.2 Hz, 1 H), 9.33 (d, *J* = 1.2 Hz, 1 H), 8.44 (d, *J* = 7.8 Hz, 1 H), 7.53 (dd, *J* = 1.2 Hz, *J*′ = 7.8 Hz, 1 H), 7.18 (d, *J* = 9 Hz, 2 H), 7.13 (td, *J* = 1.2 Hz, *J*′ = 7.2 Hz, 1 H), 6.89 (ddd, *J* = 1.2 Hz, *J*′ = 7.2 Hz, *J*″ = 13.2 Hz, 2 H), 6.61 (d, *J* = 8.4 Hz, 2 H), 5.97 (d, *J* = 5.4 Hz, 1 H), 4.75 (q, *J* = 7.2 Hz, 1 H), 4.34 (dd, *J* = 2.4 Hz, *J*′ = 9 Hz, 1 H), 3.97–3.99 (m, 1 H), 3.70 (dd, *J* = 1.8 Hz, *J*′ = 6 Hz, 4 H), 3.61 (t, *J* = 6.6 Hz, 4 H), 3.10–3.12 (m, 2 H), 2.38–2.40 (m, 1 H), 2.20–2.21 (m, 1 H), 2.03 (dd, *J* = 6 Hz, *J*′ = 7.8 Hz, 1 H), 1.92–1.93 (m, 1 H), 1.86 (dt, *J* = 2.4 Hz, *J*′ = 14.4 Hz, 1 H), 1.65–1.66 (m, 1 H), 1.48 (dq, *J* = 6.6 Hz, *J*′ = 13.2 Hz, 1 H), 1.39–1.42 (m, 4 H), 1.27–1.31 (m, 5 H), 0.95 (s, 1 H), 0.84–0.86 (m, 9 H); ^13^C NMR (150.9 MHz, CDCl_3_) δ 170.5, 162.0, 160.2, 146.2, 145.1, 142.6, 142.1, 140.1, 130.8, 127.4, 125.2, 124.5, 123.7, 119.9, 118.4, 112.2, 85.9, 77.9, 69.3, 54.4, 54.0, 53.5, 51.4, 40.5, 40.4, 40.0, 39.6, 38.2, 37.4, 35.6, 29.6, 28.6, 28.0, 27.8, 27.1, 26.3, 25.4, 24.0, 23.0, 22.0. 

*N*-((2*R*)-3-(4-(bis(2-chloroethyl)amino)phenyl)-1-(((1*R*)-3-methyl-1-((3a*S*)-3a,5,5-trimethylhexahydro-4,6-methanobenzo[*d*][1,3,2]dioxaborol-2-yl)butyl)amino)-1-oxopropan-2-yl)pyrazine-2-carboxamide (**8**): To a stirring solution of pyrazine-2-carboxylic acid (8 mg, 0.06 mmol) in DMF (100 μL), DIPEA (32 μL, 0.18 mmol) and HATU (26 mg, 0.07 mmol) were added and the mixture was cooled at 0 °C. Then, (*S*)-3-(4-(bis(2-chloroethyl)amino)phenyl)-1-(((*S*)-3-methyl-1-((3a*S*,4*S*,6*S*,7a*R*)-3a,5,5-trimethylhexahydro-4,6-methanobenzo[*d*][1,3,2]dioxaborol-2-yl)butyl)amino)-1-oxopropan-2-aminium chloride salt (30 mg, 0.05 mmol) was added and the reaction mixture was left under stirring at 0 °C for 30 min and subsequently at room temperature for 20 h. Upon completion, the mixture was subjected to FCC purification using toluene/ethyl acetate 7:3 as eluent, and the hybrid was afforded as a bright yellow oil (13 mg, 38% yield). R*f* (PhMe/EtOAc) 7:3 = 0.2; IR (KBr): 2925, 1662, 1533, 1027 cm^−1^; HR-ESI (*m*/*z*) [M+H^+^] calculated for [C_33_H_47_BCl_2_N_5_O_4_]^+^ 658.3093 Found: 658.3079; ^1^H NMR (CDCl_3_, 600.13 MHz) δ 9.35 (d, *J* = 1.8 Hz, 1 H), 8.74 (d, *J* = 2.4 Hz, 1 H), 8.53 (dd, *J* = 0.6 Hz, *J*′ = 1.8 Hz, 1 H), 8.33 (d, *J* = 8.4 Hz, 1 H), 7.17 (d, *J* = 8.4 Hz, 2 H), 6.60 (d, *J* = 8.4 Hz, 2 H), 5.95 (d, *J* = 4.8 Hz, 1 H), 4.74 (td, *J* = 1.2 Hz, *J*′ = 6.6 Hz, 1 H), 4.32 (dd, *J* = 1.8 Hz, *J*′ = 9 Hz, 1 H), 3.69 (t, *J* = 6.6 Hz, 4 H), 3.60 (t, *J* = 7.2 Hz, 4 H), 3.09–3.11 (m, 3 H), 2.33–2.34 (m, 1 H), 2.19 (ddd, *J* = 1.8 Hz, *J*′ = 6 Hz, *J*″ = 10.8 Hz, 1 H), 2.02 (t, *J* = 5.4 Hz, 1 H), 1.91 (tt, *J* = 3 Hz, *J*′ = 6 Hz, 1 H), 1.85 (dt, *J* = 2.4 Hz, *J*′ = 14.4 Hz, 1 H), 1.58–1.60 (m, 3 H), 1.48 (dq, *J* = 7.2 Hz, *J*′ = 13.8 Hz, 1 H), 1.41 (s, 3 H), 1.29 (s, 3 H), 0.82–0.86 (m, 9 H); ^13^C NMR (150.9 MHz, CDCl_3_) δ 170.7, 162.8, 147.4, 144.3, 144.1, 142.7, 130.8, 129.0, 128.2, 112.2, 85.8, 77.8, 54.2, 53.5, 51.4, 40.4, 40.0, 39.6, 38.2, 37.3, 35.6, 28.6, 27.1, 26.3, 25.4, 24.0, 23.0, 22.0.

*N*-((2*R*)-3-(4-(bis(2-chloroethyl)amino)phenyl)-1-(((1*R*)-3-methyl-1-((3a*S*)-3a,5,5-trimethylhexahydro-4,6-methanobenzo[*d*][1,3,2]dioxaborol-2-yl)butyl)amino)-1-oxopropan-2-yl)-5-chloropyrazine-2-carboxamide (**9**): To a stirring solution of 5-chloropyrazine-2-carboxylic acid (10 mg, 0.06 mmol) in DMF (100 μL), DIPEA (32 μL, 0.18 mmol) and HATU (26 mg, 0.07 mmol) were added and the mixture was cooled at 0 °C. Then, (*S*)-3-(4-(bis(2-chloroethyl)amino)phenyl)-1-(((*S*)-3-methyl-1-((3a*S*,4*S*,6*S*,7a*R*)-3a,5,5-trimethylhexahydro-4,6-methanobenzo[*d*][1,3,2]dioxaborol-2-yl)butyl)amino)-1-oxopropan-2-aminium chloride salt (30 mg, 0.05 mmol) was added and the reaction mixture was left under stirring at 0 °C for 30 min and subsequently at room temperature for 20 h. Upon completion, the mixture was subjected to FCC purification using toluene/ethyl acetate 9:1 as eluent, and the hybrid was afforded as a pale-yellow oil (15 mg, 43% yield). R*f* (PhMe/EtOAc) 8:2 = 0.17; IR (KBr): 2925, 1662, 1533, 1027 cm^−1^; HR-ESI (*m*/*z*) [M+H^+^] calculated for [C_33_H_46_BCl_3_N_5_O_4_]^+^ 692.2703 Found: 692.2691; ^1^H NMR (CDCl_3_, 600.13 MHz) δ 8.71–8.73 (m, 2 H), 8.69 (d, *J* = 1.2 Hz, 1 H), 8.51 (ddd, *J* = 1.2 Hz, *J*′ = 4.8 Hz, *J*″ = 8.4 Hz, 1 H), 8.22 (d, *J* = 7.8 Hz, 1 H), 7.50 (ddd, *J* = 4.2 Hz, *J*′ = 5.4 Hz, 1 H), 7.15 (d, *J* = 9 Hz, 2 H), 6.60 (d, *J* = 8.4 Hz, 2 H), 5.98 (d, *J* = 5.4 Hz, 1 H), 4.70–4.71 (m, 1 H), 4.33 (dd, *J* = 2.4 Hz, *J*′ = 9 Hz, 1 H), 3.80–3.82 (m, 1 H), 3.70 (t, *J* = 6.6 Hz, 4 H), 3.61 (t, *J* = 7.2 Hz, 4 H), 3.09–3.11 (m, 2 H), 2.35 (ddt, *J* = 3 Hz, *J*′ = 5.4 Hz, *J*″ = 11.4 Hz, 1 H), 2.19–2.21 (m, 1 H), 2.02 (dt, *J* = 5.4 Hz, *J*′ = 6 Hz, 1 H), 1.92 (ddd, *J* = 3 Hz, 1 H), 1.85 (ddd, *J* = 1.2 Hz, *J*′ = 15 Hz, 1 H), 1.42 (s, 2 H), 1.38–1.39 (m, 1 H), 1.30–1.32 (m, 3 H), 1.25–1.27 (m, 1 H), 0.83–0.87 (m, 9 H); ^13^C NMR (150.9 MHz, CDCl_3_) δ 170.6, 161.6, 160.6, 152.0, 145.1, 142.1, 141.7, 140.6, 135.1, 130.7, 129.8, 125.2, 121.1, 112.1, 85.8, 65.5, 54.2, 53.5, 51.4, 40.4, 40.0, 39.6, 38.2, 37.4, 35.6, 28.6, 28.0, 27.1, 26.3, 25.4, 24.0, 23.0, 22.0.

### 3.3. Cell Lines and Culture Conditions

The multiple myeloma human plasma cells RPMI 8226 were purchased from American Type Culture Collection (ATCC, Manassas, VA, USA) and cultured in RPMI 1640 medium (Gibco, Paisley, UK) supplemented with 50 µg/mL streptomycin sulfate, 50 U/mL penicillin G sodium salt (Gibco, Grand Island, NY, USA) and 10% fetal bovine serum (FBS) (Biowest, Nuaillé, France). RPMI 8226/BTZ100 cells were kindly provided by Dr. Jacqueline Cloos (VU University Medical Center, Amsterdam, The Netherlands) and kept in culture in the same conditions described previously for RPMI 8226 with the addition of 100 nM BTZ at the time of each splitting. Human MSC from the bone marrow were purchased from PromoCell and were cultured in Mesenchymal Stem Cell Growth Medium 2 (PromoCell, Heidelberg, Germany) supplemented with 100 μg/mL streptomycin sulfate and 100 U/mL penicillin G sodium salt.

### 3.4. 3D Co-Culture Spheroids 

RPMI 8226 cells and MSC were mixed in a 5:1 ratio. The cell mixture was centrifuged, and medium containing 20% collagen I (Gibco) adjusted to pH 7 was added to the cell pellet. Five μL drops were plated in each well of an ice-cold 96-well ultra-low attachment U-bottom plate (Corning, Kennebunk, MA, USA) and the plate was incubated for 30 min at 37 °C and 5% CO_2_ atmosphere. After the incubation, 200 μL of RPMI 1640 medium (Gibco) supplemented with 50 µg/mL streptomycin sulfate, 50 U/mL penicillin G sodium salt (Gibco) and 10% FBS (Biowest) was added to each well. The 3D spheroids were formed after 48 h.

### 3.5. Antiproliferative Assay

RPMI 8226 and RPMI 8226/BTZ100 cells were seeded in 96-well plates (Costar, Kennebunk, MA, USA) at a density of 15,000 cells per well and directly treated for 72 h with the various compounds using increasing concentrations (0.31–2000 nM). BTZ was used as a positive control and dimethyl sulfoxide (DMSO) as the vehicle control (0.1% in culture medium). At the end of the treatment time, cells were incubated with 50 µL of an XTT solution (2,3-bis-(2-methoxy-4-nitro-5-sulfophenyl, 15 mg/mL) (Sigma, St. Louis, MO, USA) for 4 h. The absorbance was red at 450 nm using a Cytation 3 plate reader (BioTek, Winooski, VT, USA). The percentage of cell proliferation was measured by comparing the absorbance of the sample treated with the vehicle control wells and multiplied by 100. The IC_50_ value was calculated through a nonlinear regression of 7 different concentrations using GraphPad Prism 9 (San Diego, CA, USA). Spheroids were treated for 48 h with the compounds having shown an IC_50_ value < 50 uM in RPMI 8226 cells using increasing concentrations (12.5–800 nM). Following the treatment, 100 μL of medium was carefully removed from each well and collagenase (1.5 μL of a 100 mg/mL solution, Sigma) was added. The plate was shaken at 37 °C for 10 min at 350 rpm. Then, 100 μL of CellTiter-Glo 3D reagent (Promega, Fitchburg, WI, USA) was added to each well. The plate was incubated for 5 min at 37 °C under agitation at 250 rpm, followed by 25 min at room temperature without agitation. Finally, the luminescence was measured using a Cytation 3 plate reader (BioTek). The percentage of cell proliferation and calculation of the IC_50_ value were determined as previously described. Each compound was tested in triplicate in three independent experiments.

### 3.6. Proteasome Inhibitory Activity

RPMI 8226 cells (1 × 10^6^ cells/mL) were collected, centrifuged, and washed twice with cold PBS (Gibco). The supernatant was discarded, and 200 μL of ice-cold lysis buffer (NP40 cell lysis buffer (Invitrogen, Eugene, OR, USA), supplemented with 1 mM PMSF (Sigma) and protease inhibitor cocktail (Sigma), was added to the pellet. Cells were kept on ice for 30 min and vortexed every 10 min. Samples were then centrifuged at 4 °C for 10 min at 16,000× *g*. Supernatant containing the proteins was collected. Protein quantification was performed using the Coomassie (Bradford) Protein Assay Kit (ThermoFisher, Waltham, MA, USA) following the manufacturer instructions. In each well of a 96-well black plate (ThermoFisher), 10 µg of proteins were mixed with assay buffer (50 mM Tris-HCl, pH 7.5, 40 mM KCl, 5 mM MgCl_2_, 0.5 mM ATP, 1 mM DTT, 0.5 mg/mL BSA) in 180 µL total volume. The compounds were diluted in assay buffer and tested at 7 different concentrations (0.78–50 nM) with a final concentration of DMSO that did not exceed 1.5%. The plate was then incubated for 30 min at 37 °C. After incubation, the reaction was started by adding the fluorometric peptide substrate Suc-LLVY-amc (Bio-Techne, Minneapolis, MN, USA) at a final concentration of 100 µM, except for the blank wells. Following 1 h incubation at 37 °C in the dark, the fluorescence signal was red (excitation: 380 nm, emission: 440–460 nm) using a Cytation 3 plate reader (BioTek) [19]. The percentage of 26S proteasome activity was measured by comparing the fluorescence of the sample treated with the vehicle control wells and multiplied by 100. The IC_50_ value was calculated as mentioned above, and each compound was tested in triplicate in three independent experiments.

### 3.7. HDAC Inhibitory Activity

RPMI 8226 cells were seeded in 96-well plates (Costar) at a density of 15,000 cells per well in 25 µL of medium containing the HDAC substrate *N*-(4-methyl-7-aminocoumarinyl)-*N*α-(t-butoxycarbonyl)-*N*ω-acetyllysineamide (21 µM, MAL, Sigma). The cells were directly treated with the compounds at 10 µM or with Ent (0.4–250 µM) for 8 h to obtain a final volume of 50 µL. The final percentage of DMSO did not exceed 0.5%. Following the incubation, the reaction was stopped by adding 10 µL of 6_X_ RIPA buffer, supplemented with protease inhibitor (Sigma). Then, 160 µL of cold acetonitrile were added and the plate was placed for 10 min at −80 °C. The plate was centrifuged at 5000× *g* for 10 min at 4 °C, and the supernatant was transferred into a UHPLC-adapted 96-well plate (Nunc 96-well plate conical bottom, ThermoFisher) and sealed. The substrate (MAL) and its deacetylated product (dMAL) were measured using a UHPLC-MS method as previously described [20]. The percentage of HDAC inhibition is given by 100 − [(tested sample × 100/control sample)], and the dose-response curve of Ent was established.

### 3.8. Statistical Analysis

Statistical analysis was performed in GraphPad Prism 9 (GraphPad) using one-way ANOVA for the proteasome inhibition assay. Combenefit (Cancer Research UK Cambridge Institute, Cambridge, UK) and CompuSyn software were used for drug combination analyses. The CI was calculated through the Chou-Talalay method using Compusyn. Data are expressed as mean ± standard deviation (SD).

## 4. Conclusions

In conclusion, from a total of eleven histone deacetylase-proteasome inhibitor hybrids sharing in common the 1,2-dianiline carboxamide type zinc-binding group of Ent and the phenylalanine-boroleucine pharmacophore of BTZ, two of them, compounds **2** and **3**, exhibited the strongest antiproliferative activity against both RPMI 8226 and RPMI/BTZ100 (resistant to BTZ) multiple myeloma cell lines. The improved activity of the hybrids **2** and **3**, after taking into account the so far structure–activity analysis, may be attributed to the spacer tethering the two above-mentioned pharmacophores, thus the terephthalic acid (in case of compound **2**) and the 2,5-pyrazinedicarboxylic acid (in case of compound **3**), which offers them a shorter chain and more rigid structure compared to the spacers of the other hybrid compounds. The latter, due to the presence of 4-methylamino benzoyl or 4-carboxy-phenylacetyl moieties, have a longer chain and more flexible structures. On the other hand, the attempt to incorporate a cytotoxic “warhead” in compounds **8**, **9**, and **11** by replacing the phenylalanine moiety with melphalan failed to offer the presumed improvement of activity. Based on this work, compounds with improved activity against bortezomib-resistant cells may be designed.

## Figures and Tables

**Table 1:** Activity of compounds **1**–**11** in various MM models (IC_50_ values ± SD, *n* = 3).

Compound	IC_50_ Value [nM]			
	Antiproliferative Activity			PI Activity
	RPMI 8226	RPMI 8226/BTZ100	Spheroids	
**1**	677.7 ± 42.5	ND ^a^	ND	10.3 ± 2.7
**2**	37.1 ± 2.9	112.3 ± 13.0	32.5 ± 7.5	9.5 ± 2.3
**3**	9.5 ± 1.4	157.7 ± 15.8	13.1 ± 6.9	27.2 ± 6.2 *^,b^
**4**	802.4 ± 72.7	ND	ND	>50
**5**	25.2 ± 2.2	203.0 ± 36.1	65.1 ± 14.1	14.8 ± 2.5 *
**6**	336.6 ± 49.4	ND	ND	>50
**7**	31.4 ± 1.7	831.3 ± 139.3	89.3 ± 13.6	7.2 ± 1.7
**8**	19.6 ± 0.4	1008.3 ± 88.6	30.0 ± 1.6	12.1 ± 1.1
**9**	37.6 ± 1.1	1033.0 ± 205.4	188.2 ± 42.5	>50
**10**	>2000	ND	ND	>50
**11**	22.9 ± 2.6	462.0 ± 107.4	39.9 ± 12.1	>50
**Ent**	328.9 ± 25.7	561.7 ± 51.7	348.9 ± 133.2	ND
**BTZ**	2.1 ± 0.4	245.3 ± 11.1	6.4 ± 1.6	6.6 ± 0.7

^a^ ND not determined; ^b^ Significantly different values compared to BTZ as determined by one-way ANOVA * *p* < 0.05.

## Data Availability

Not applicable.

## Supplementary Materials

The following supporting information can be downloaded at: https://www.mdpi.com/article/10.3390/molecules28031456/s1, Figure S1–S20: Copies of 1H and ^13^C NMR spectra of compounds.

## Sample Availability

Samples of the compounds are not available from the authors.
