# Supplementary material for: Entinostat-Bortezomib Hybrids against Multiple Myeloma
Source: Molecules. 2023 Feb 2;28(3):1456. doi: 10.3390/molecules28031456 (PMC9920246; doi:10.3390/molecules28031456)
Supplement: Supplementary file 1 [file molecules-28-01456-s001.zip › molecules-2128592-supplementary.pdf]

SUPPORTING INFORMATION

Entinostat-bortezomib hybrids against multiple myeloma

Angelica Ferro [1,2,†], Dafni Graikioti [3,†], Emre Gezer [1,2], Constantinos M. Athanassopoulos [3,*], Muriel Cuendet [1,2,*]

[1] *School of Pharmaceutical Sciences, University of Geneva, 1211 Geneva, Switzerland*
[2] *Institute of Pharmaceutical Sciences of Western Switzerland, University of Geneva, 1211 Geneva, Switzerland* [3] *Synthetic Organic Chemistry Laboratory, Department of Chemistry, University of Patras, 26504 Patras, Greece*
* Correspondence: muriel.muendet@unige.ch (M.C.); kath@upatras.gr (C.M.A.)
† These authors contributed equally to this work.

Copies of NMR spectra

Table of contents

Figure S1. ^{1}H-NMR spectrum of compound 1. 3

Figure S2. ^{13}C-NMR spectrum of compound 1. 3

Figure S3. ^{1}H-NMR spectrum of compound 3. 4

Figure S4. ^{13}C-NMR spectrum of compound 3. 4

Figure S5. ^{1}H-NMR spectrum of compound 4. 5

Figure S6. ^{13}C-NMR spectrum of compound 4. 5

Figure S7. ^{1}H-NMR spectrum of compound 5. 6

Figure S8. ^{13}C-NMR spectrum of compound 5. 6

Figure S9. ^{1}H-NMR spectrum of compound 6. 7

Figure S10. ^{13}C-NMR spectrum of compound 6. 7

Figure S11. ^{1}H-NMR spectrum of compound 7. 8

Figure S12. ^{13}C-NMR spectrum of compound 7. 8

Figure S13. ^{1}H-NMR spectrum of compound 8. 9

Figure S14. ^{13}C-NMR spectrum of compound 8. 9

Figure S15. ^{1}H-NMR spectrum of compound 9. 10

Figure S16. ^{13}C-NMR spectrum of compound 9. 10

Figure S17. ^{1}H-NMR spectrum of compound 10. 11

Figure S18. ^{13}C-NMR spectrum of compound 10. 11

Figure S19. ^{1}H-NMR spectrum of compound 11. 12

Figure S20. ^{13}C-NMR spectrum of compound 11. 12

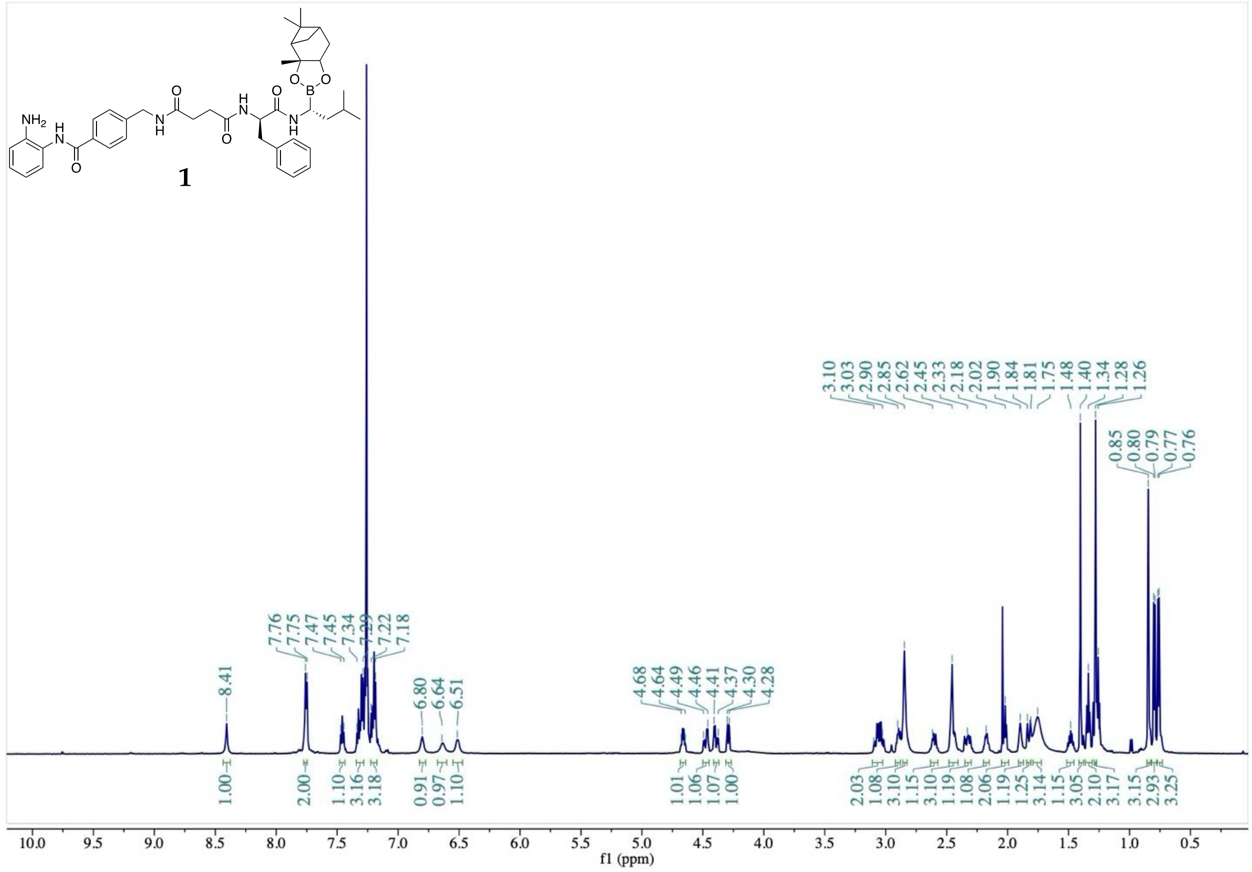

Figure S1. ^{1}H-NMR spectrum of compound 1.

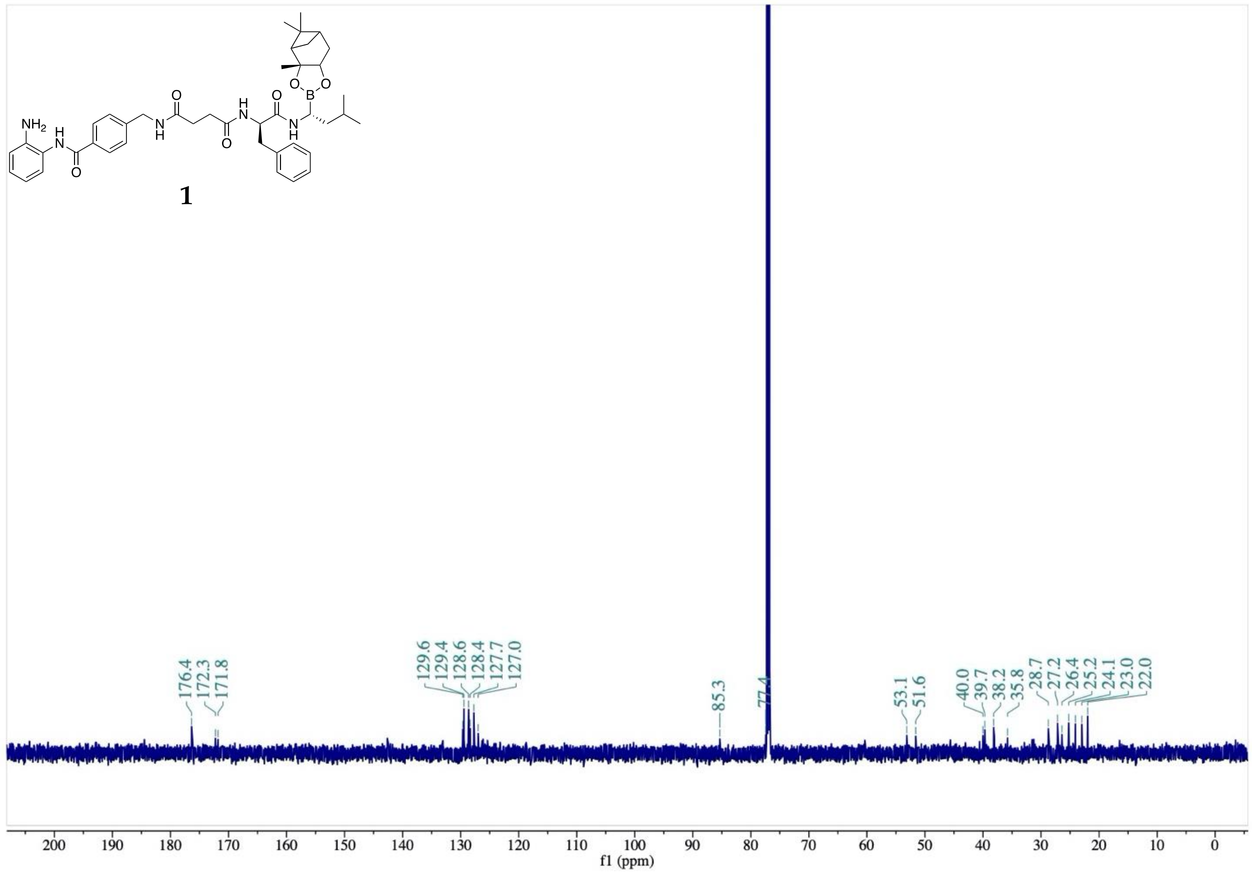

Figure S2. ^{13}C-NMR spectrum of compound 1.

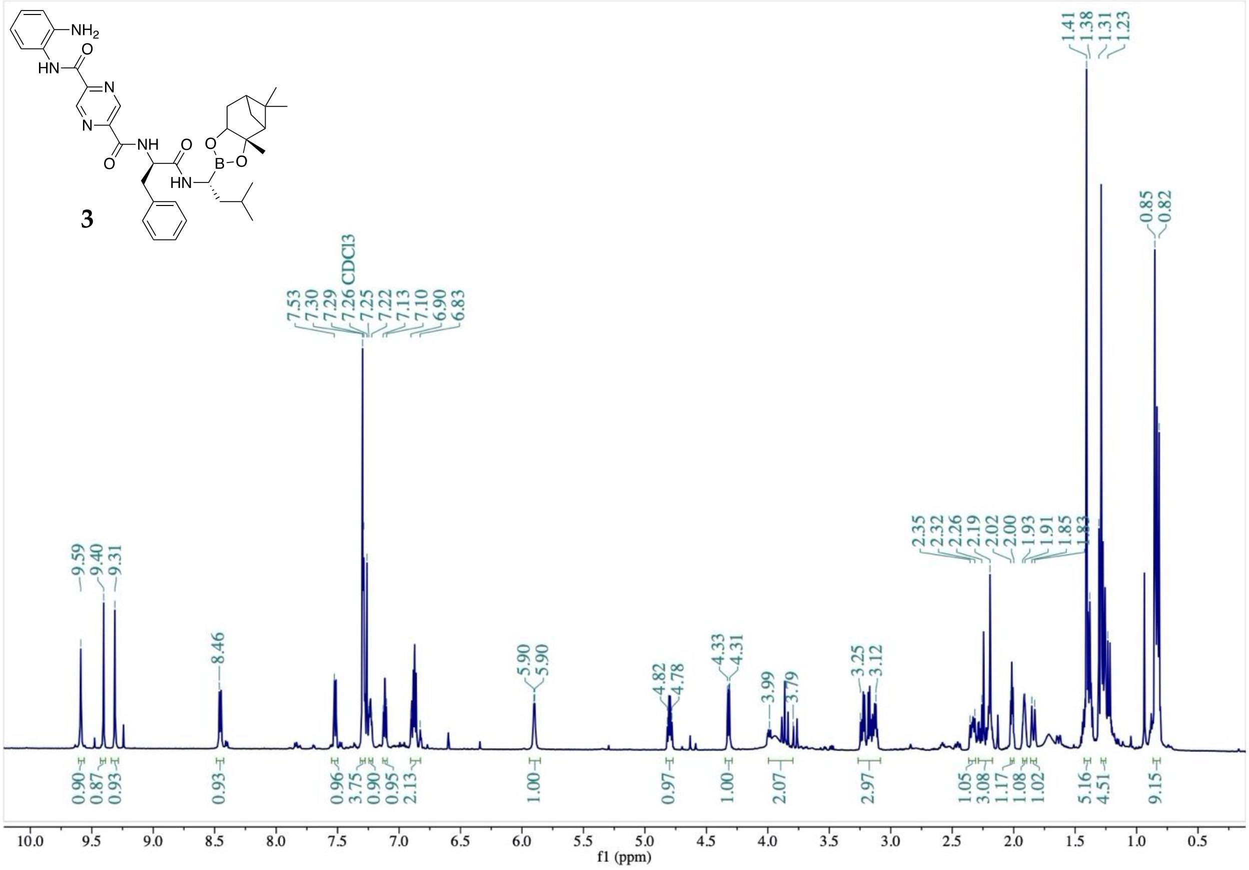

Figure S3. ^{1}H-NMR spectrum of compound 3.

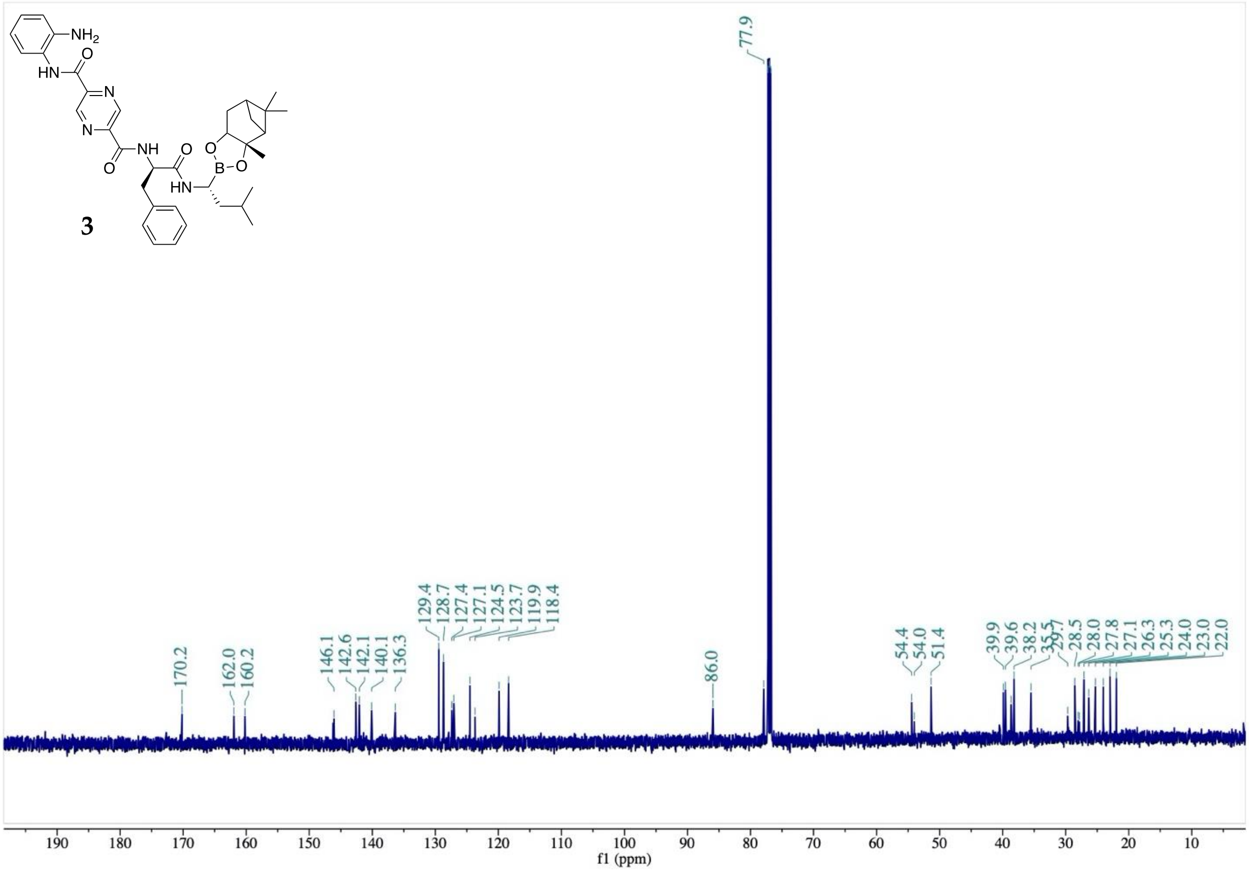

Figure S4. ^{13}C-NMR spectrum of compound 3.

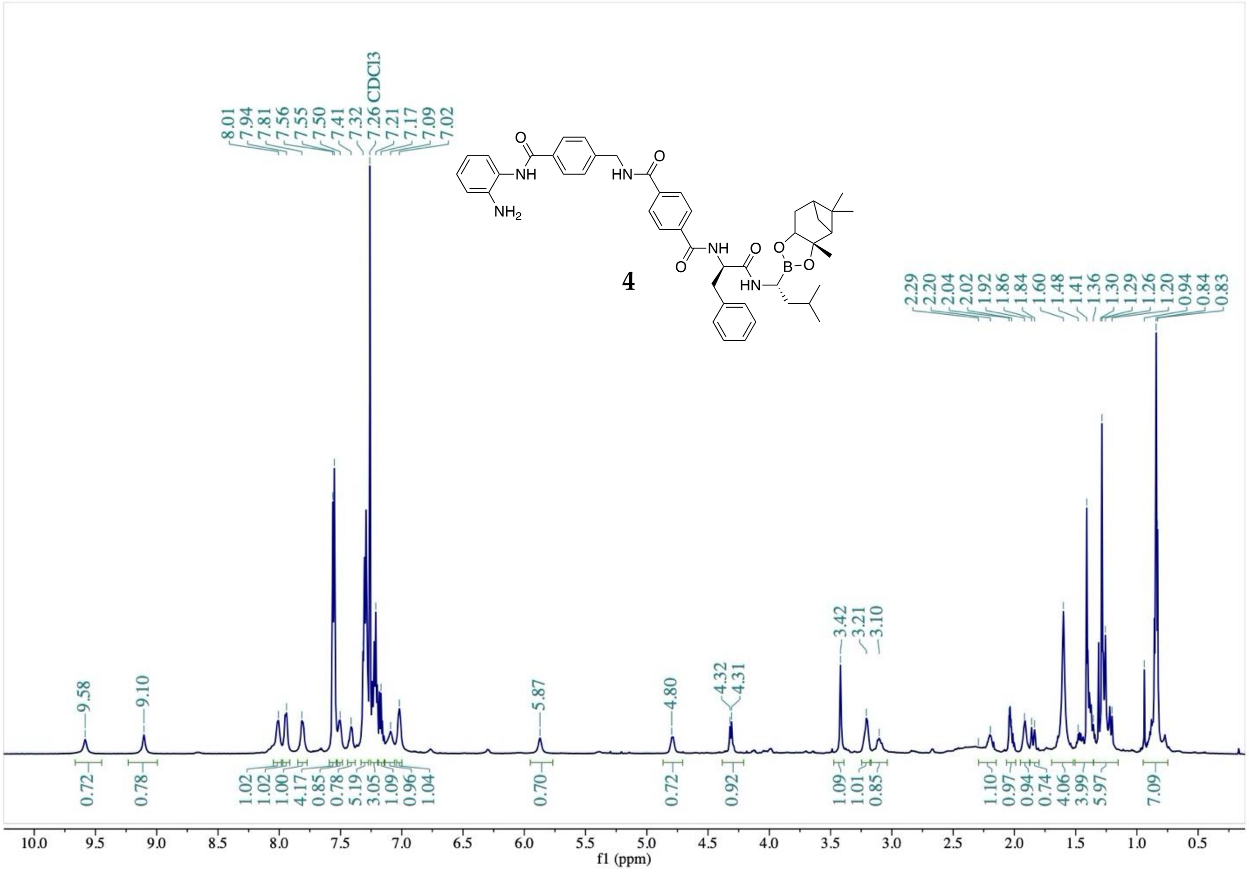

Figure S5. ^{1}H-NMR spectrum of compound 4.

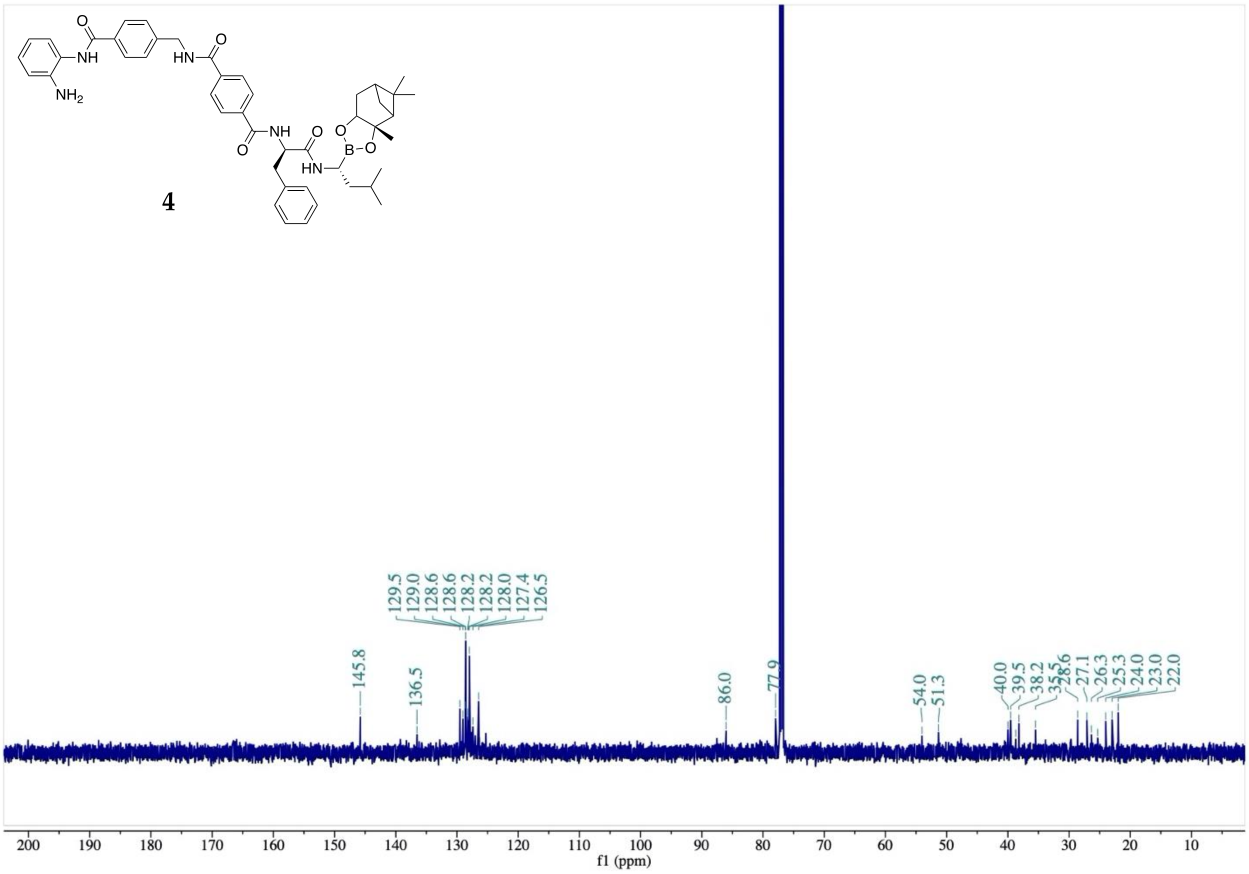

Figure S6. ^{13}C-NMR spectrum of compound 4.

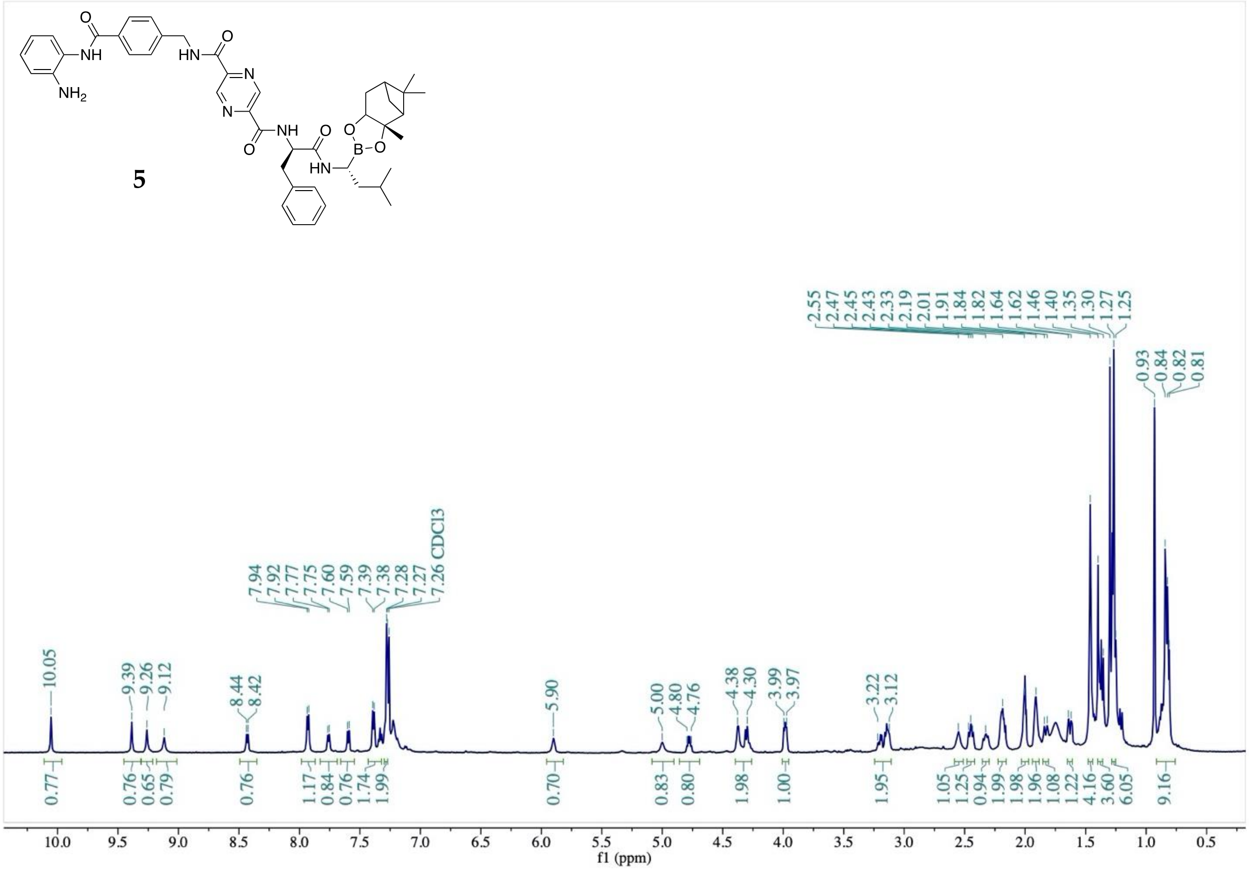

Figure S7. ^{1}H-NMR spectrum of compound 5.

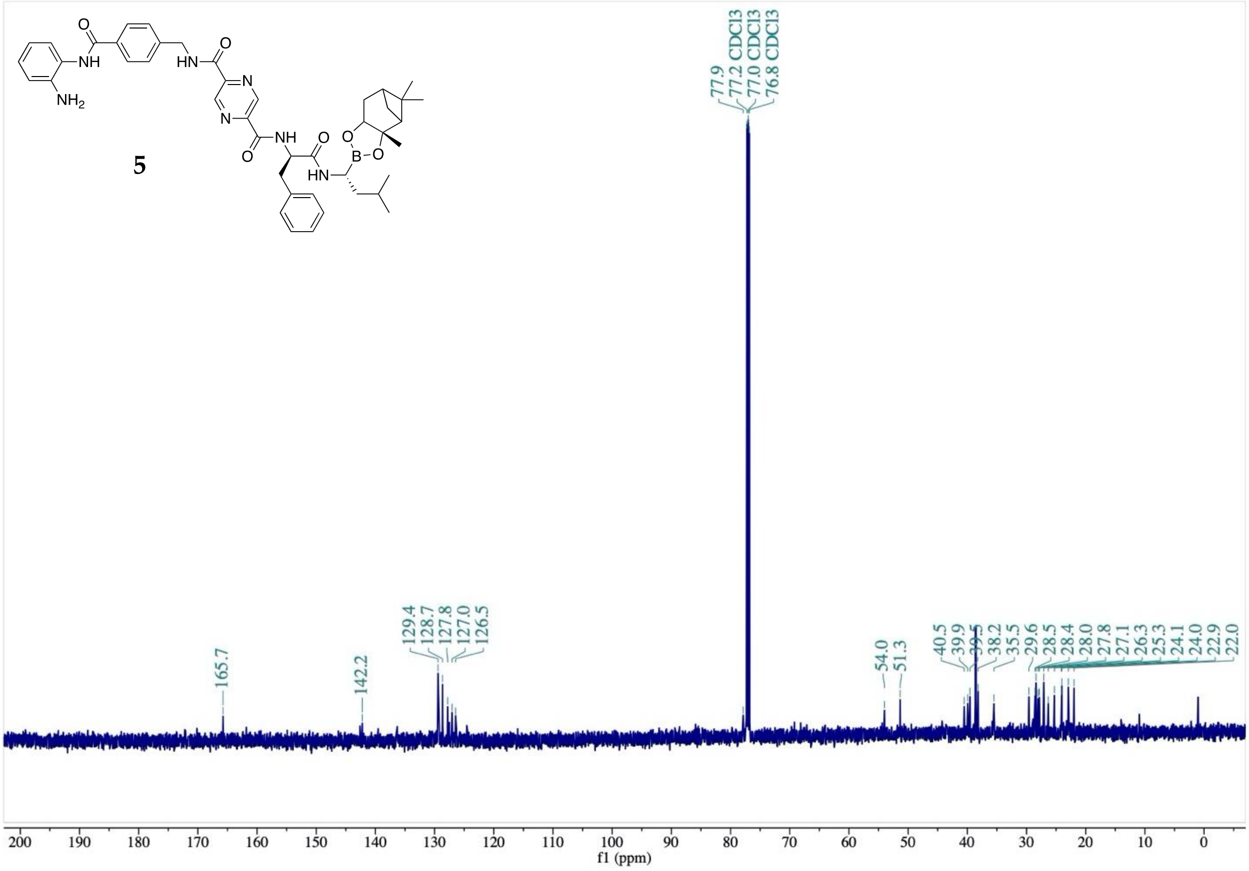

Figure S8. ^{13}C-NMR spectrum of compound 5.

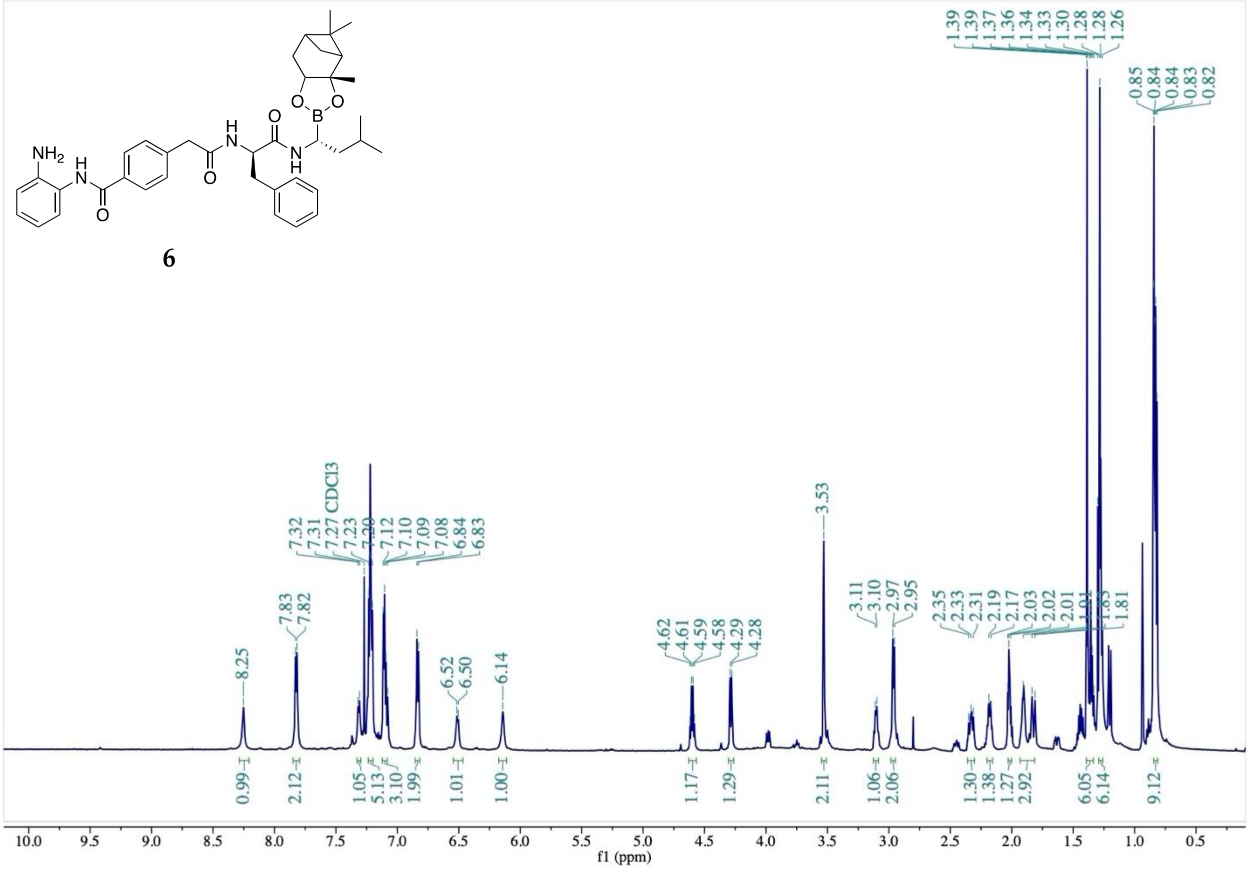

Figure S9. ^{1}H-NMR spectrum of compound 6.

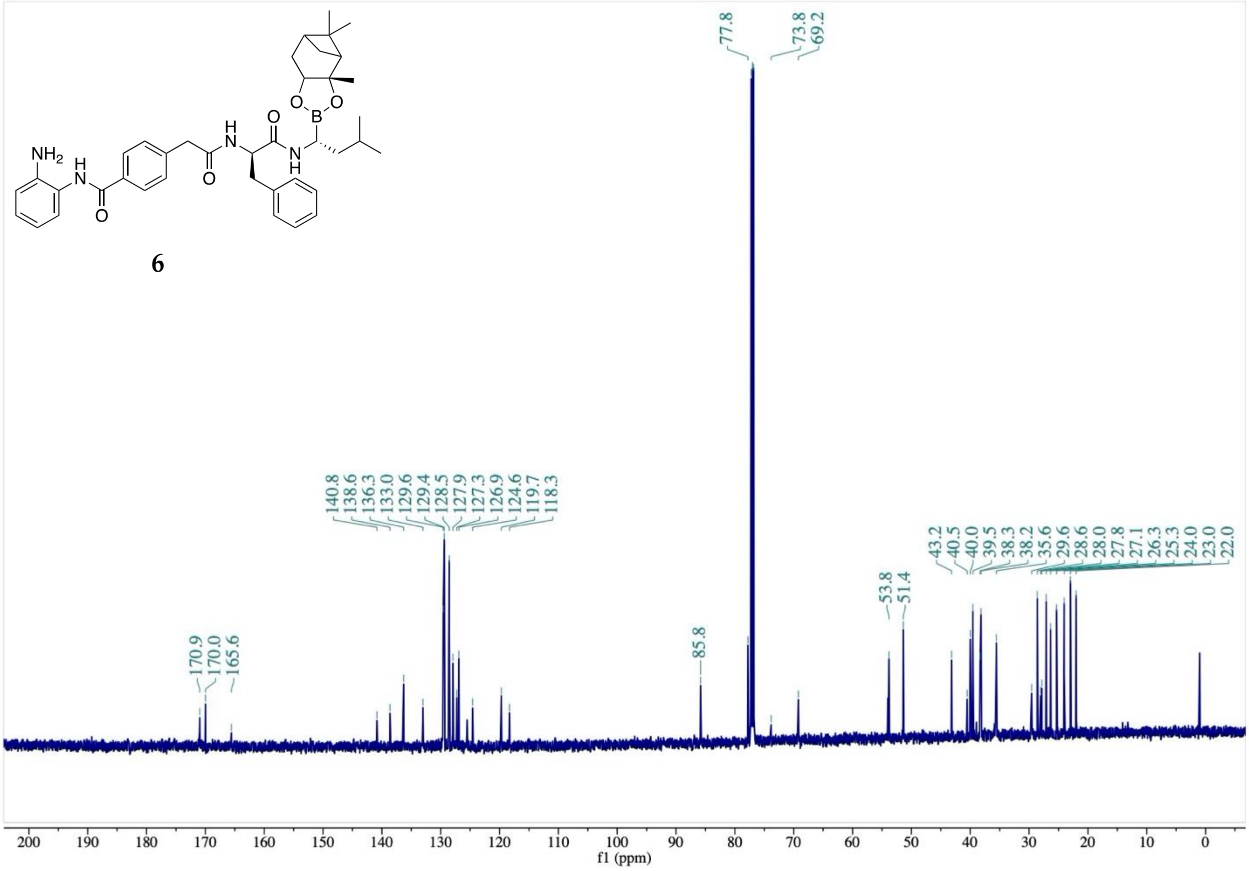

Figure S10. ^{13}C-NMR spectrum of compound 6.

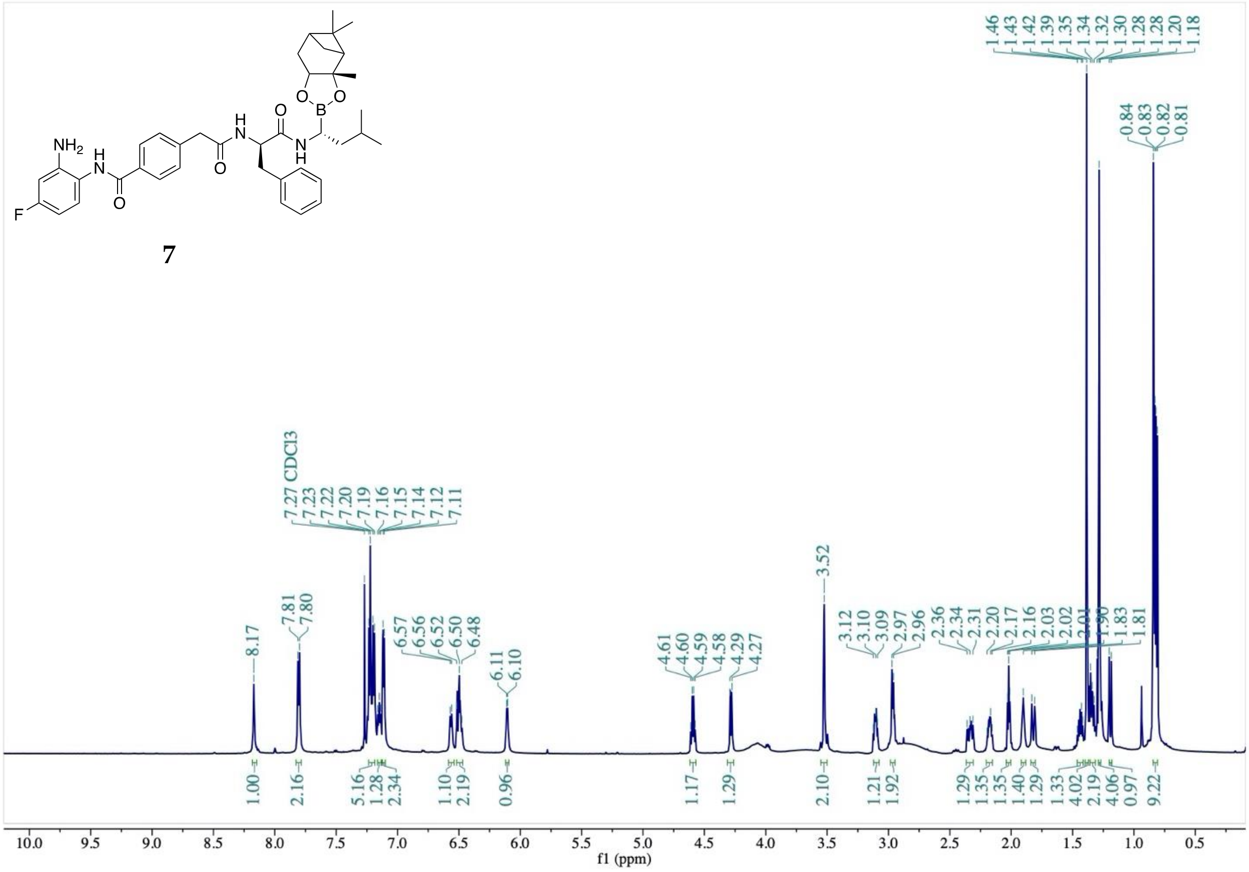

Figure S11. ^{1}H-NMR spectrum of compound 7.

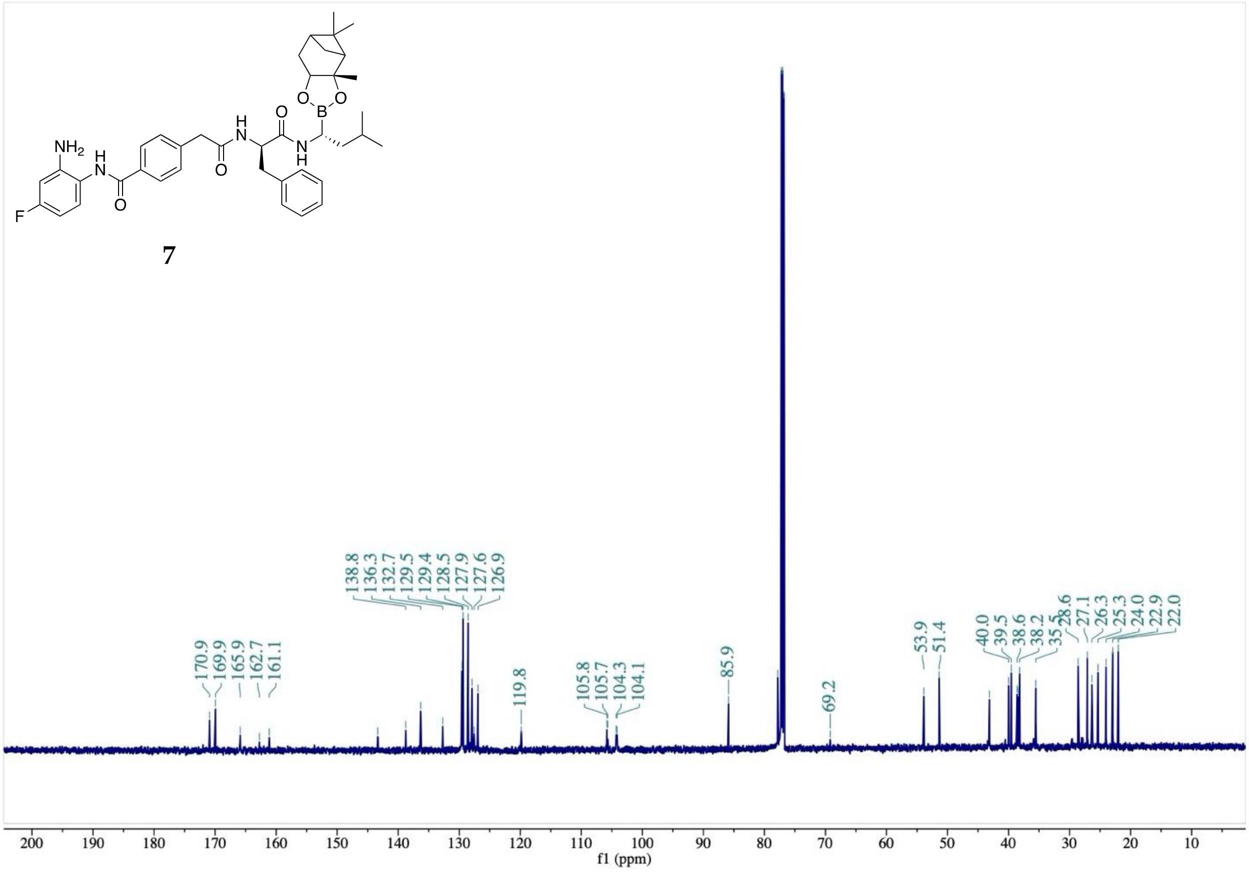

Figure S12. ^{13}C-NMR spectrum of compound 7.

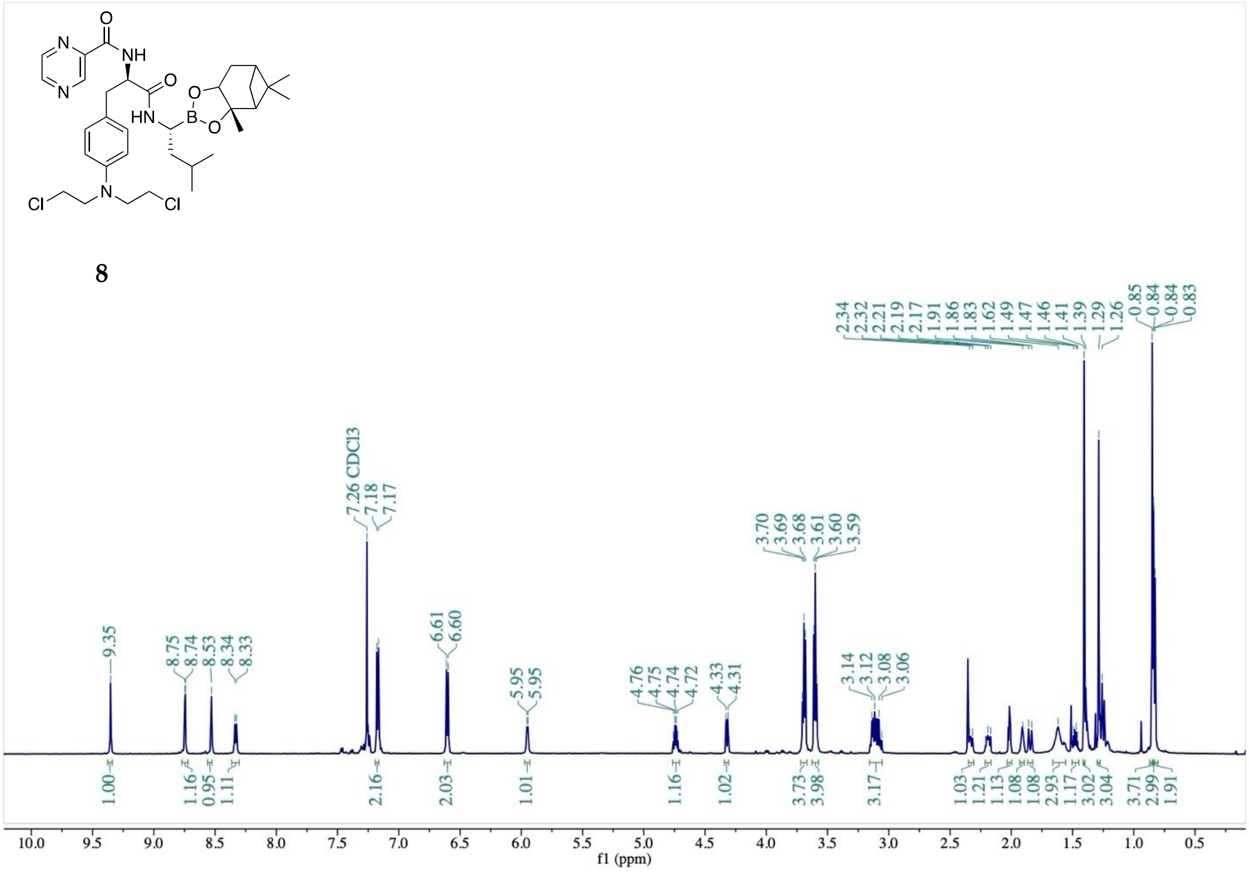

Figure S13. **^{1}H-NMR spectrum of compound 8.**

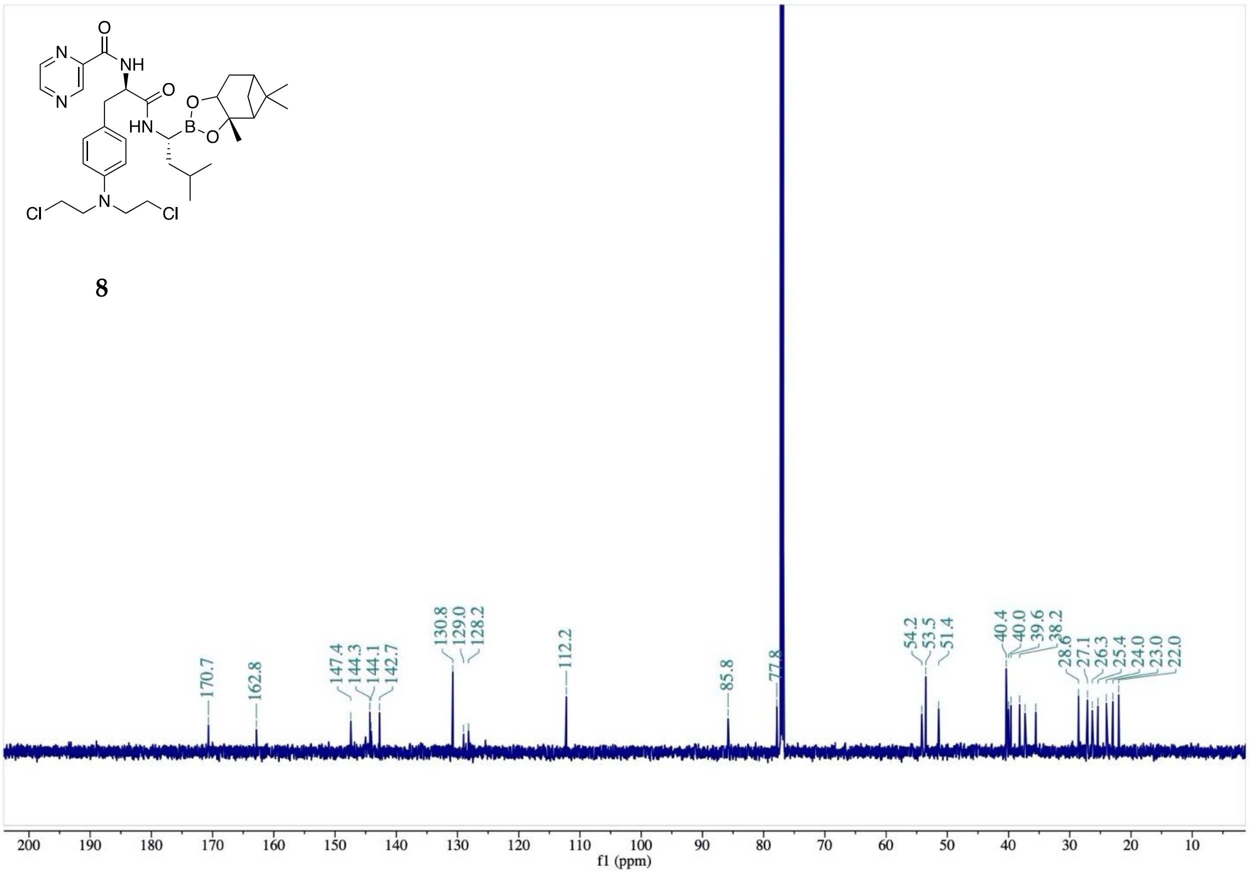

Figure S14. **^{13}C-NMR spectrum of compound 8.**

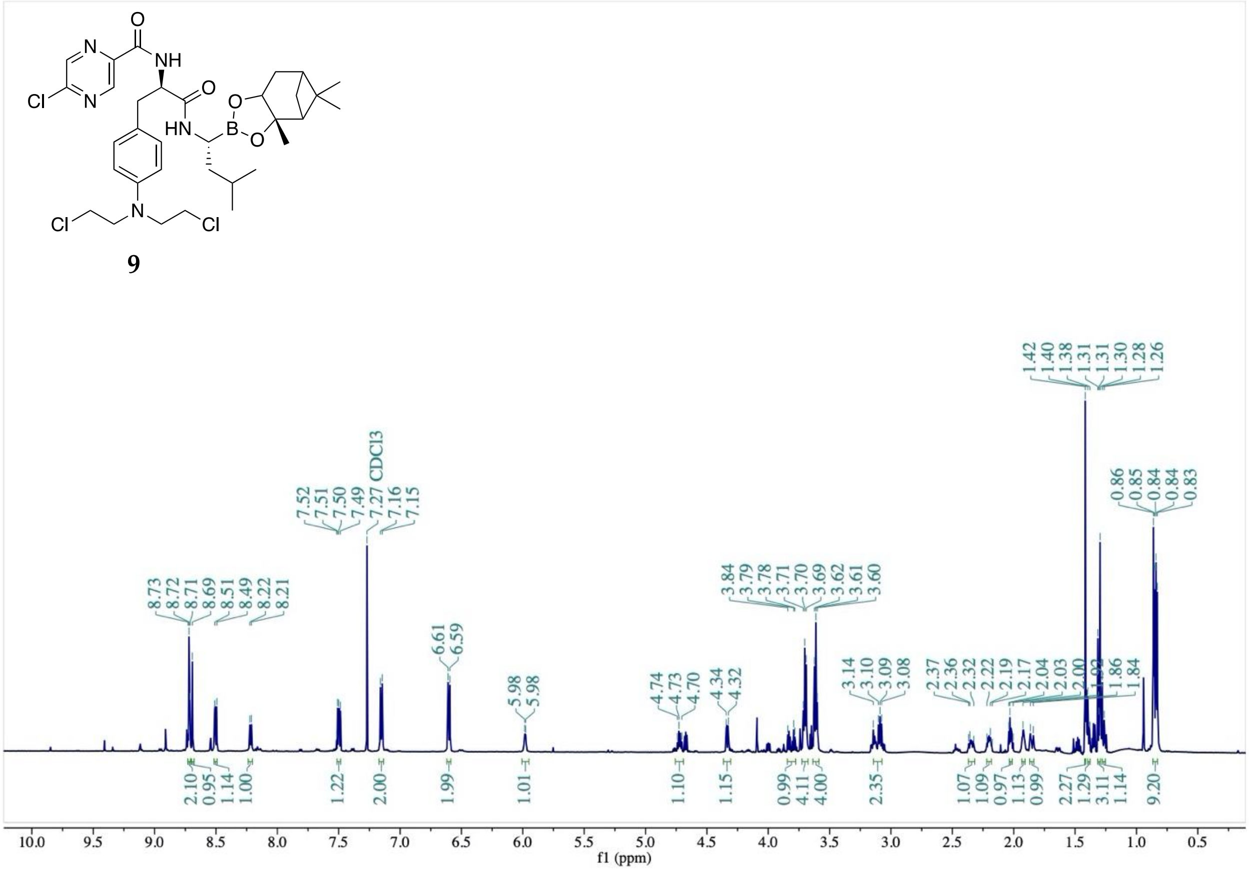

Figure S15. ^{1}H-NMR spectrum of compound 9.

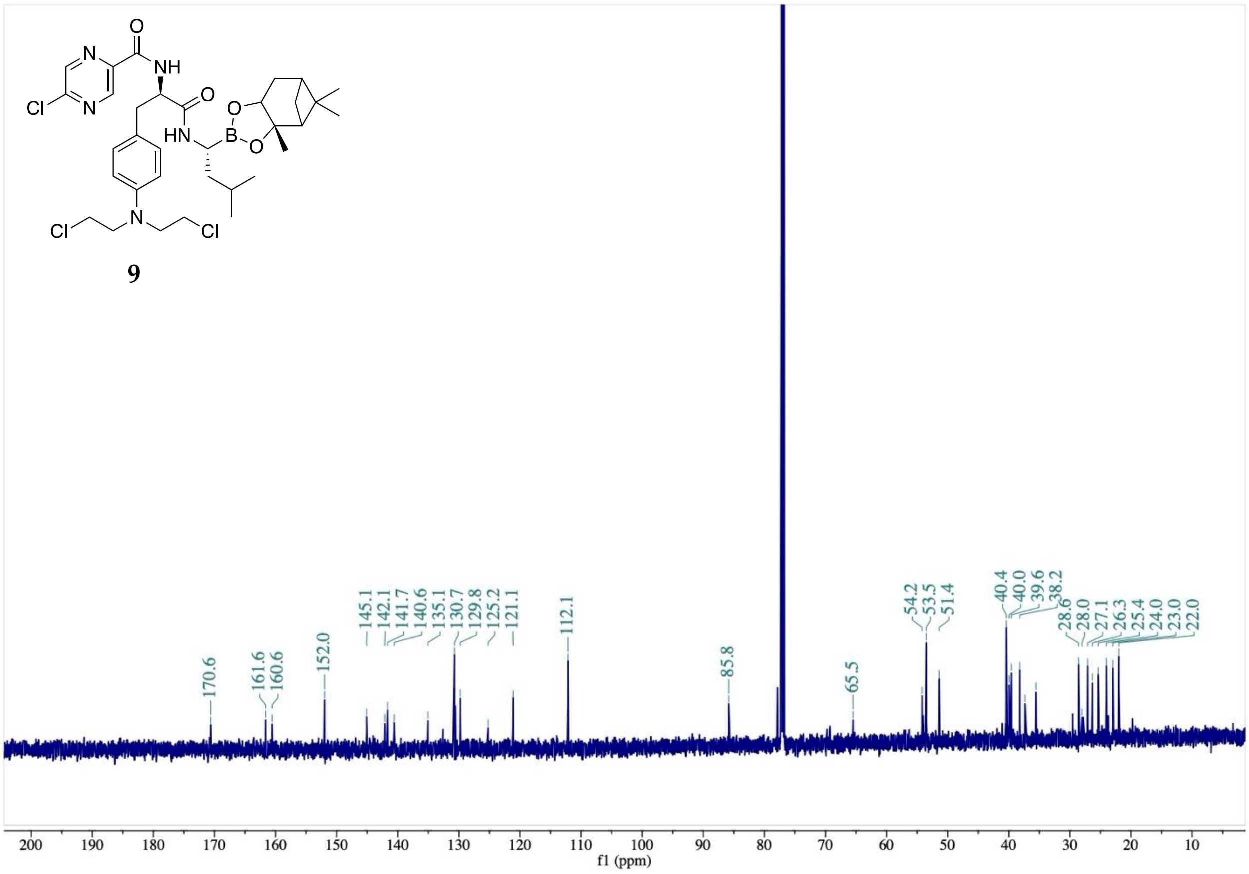

Figure S16. ^{13}C-NMR spectrum of compound 9.

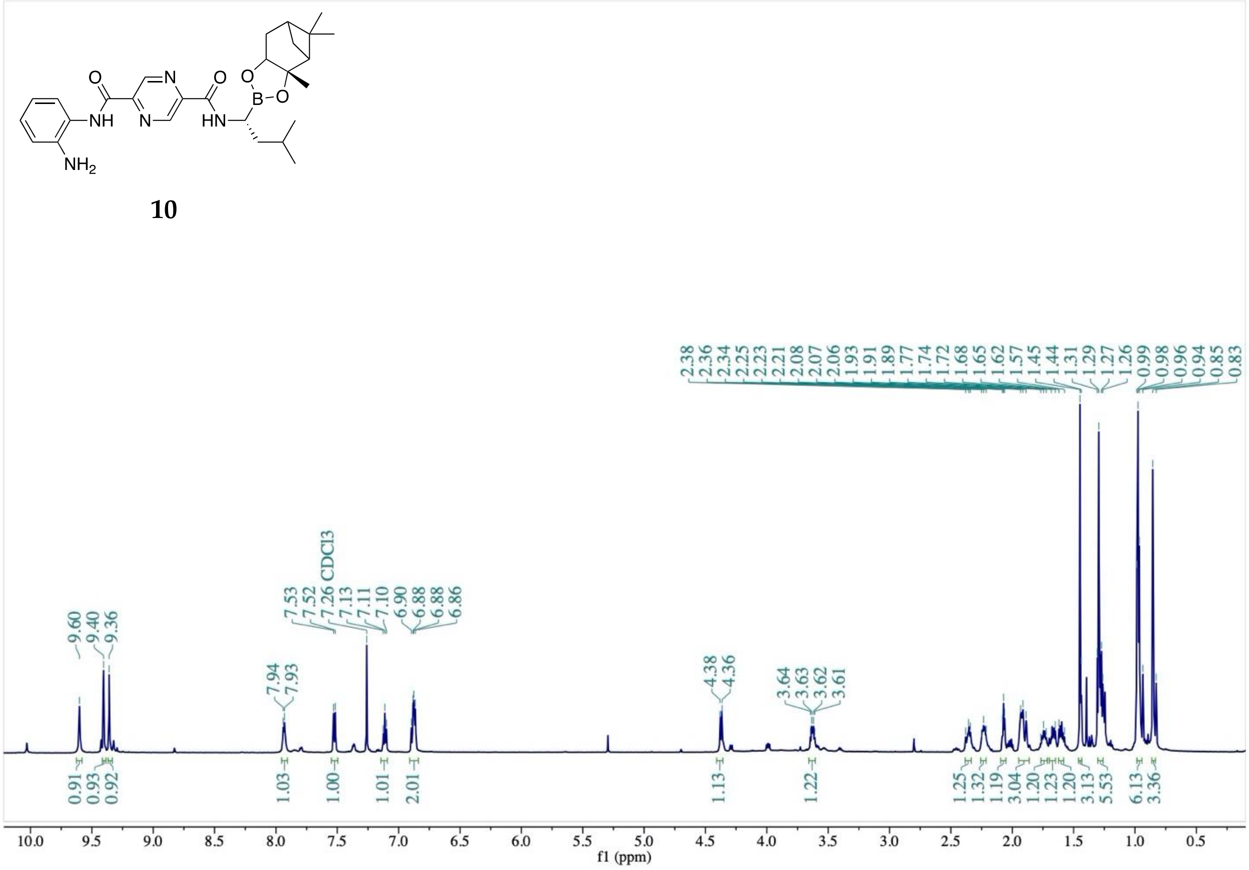

Figure S17. ^{1}H-NMR spectrum of compound 10.

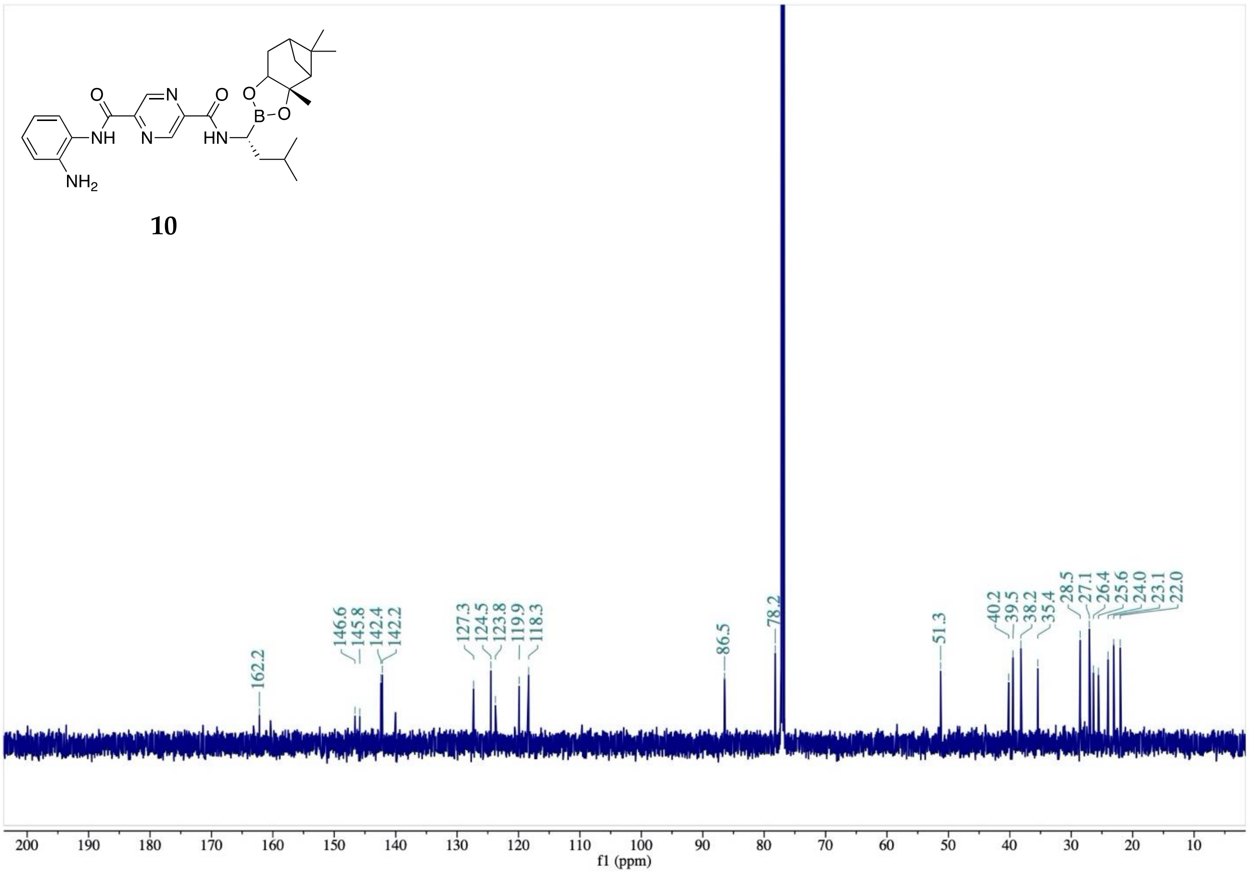

Figure S18. ^{13}C-NMR spectrum of compound 10.

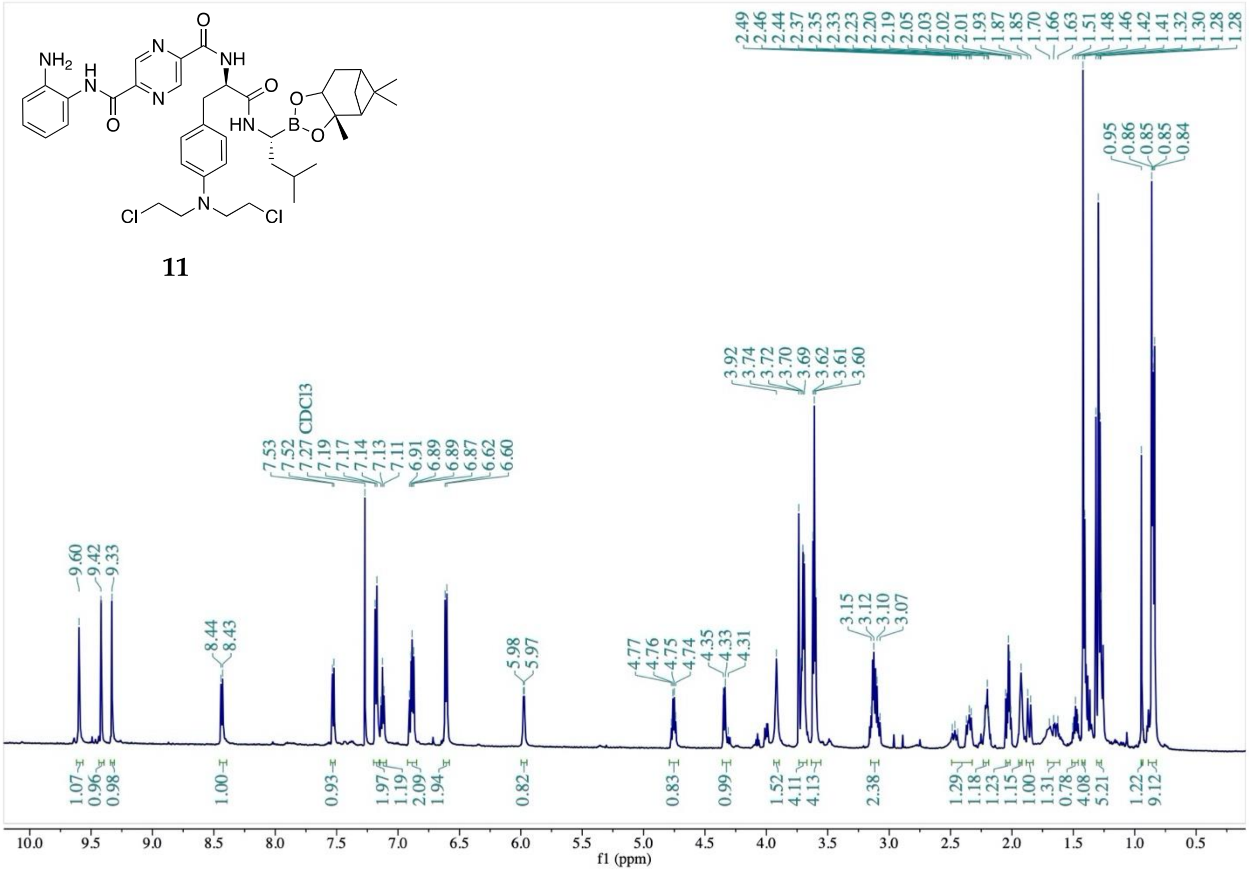

Figure S19. ^{1}H-NMR spectrum of compound 11.

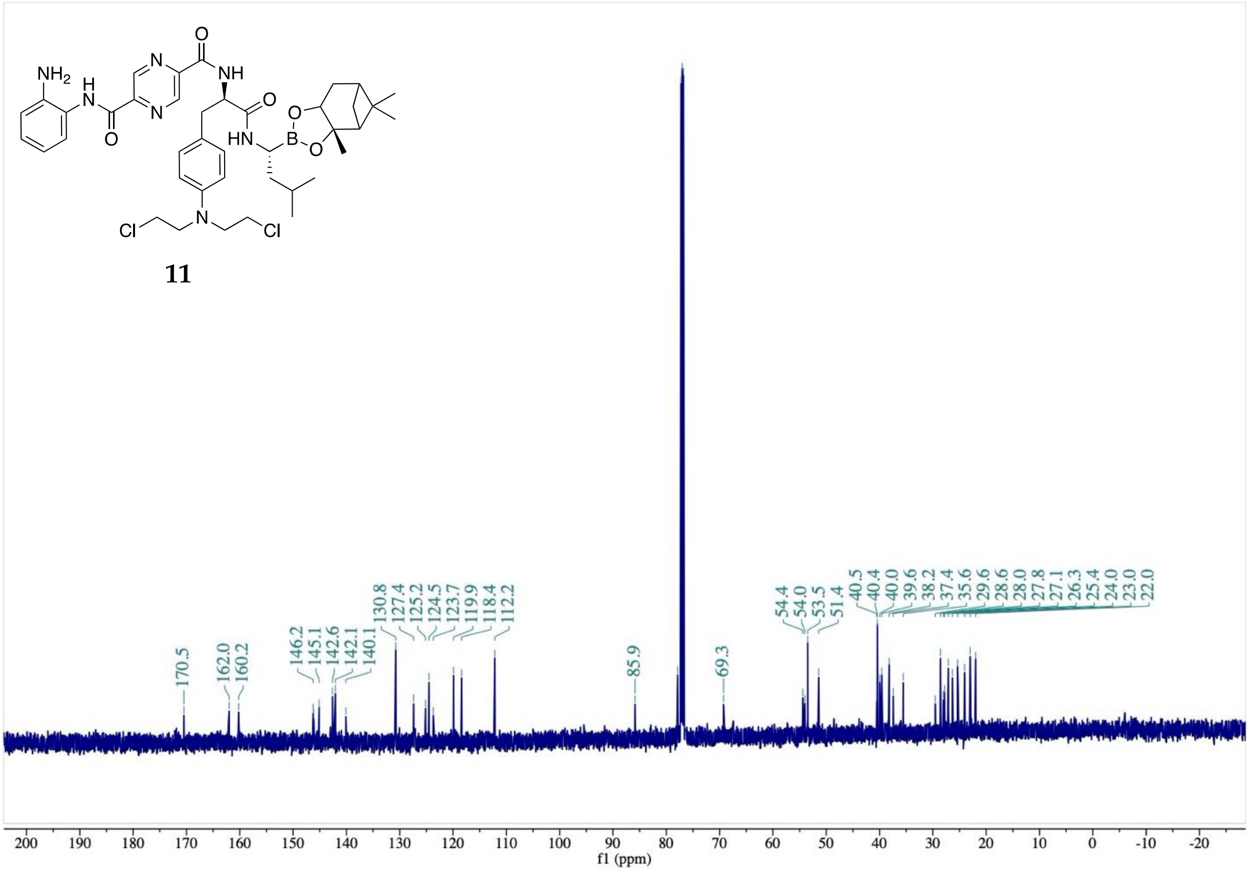

Figure S20. ^{13}C-NMR spectrum of compound 11.
